# C16orf74 is a novel prognostic biomarker and associates with immune infiltration in head and neck squamous cell carcinoma

**DOI:** 10.1371/journal.pone.0322701

**Published:** 2025-05-07

**Authors:** Xiang-Rong Yao, Fang-Zhu Xiao, Wen-Tao Xiao, Cui-Qin Huang, Jun-Yan He

**Affiliations:** 1 Department of Oncology, The First Affiliated Hospital, Hengyang Medical School, University of South China, Hengyang, China; 2 School of Public Health, University of South China, Hengyang, China; 3 Department of Radiation Oncology, Affiliated Hospital of Nantong University, Medical School of Nantong University, Nantong University, Nantong, China; 4 Department of Pathology, The First Affiliated Hospital, Hengyang Medical School, University of South China, Hengyang, China; Shanghai Jiao Tong University, CHINA

## Abstract

Head and neck squamous cell carcinoma (HNSC) is a prevalent and aggressive malignancy with poor prognosis, underscoring the need for novel biomarkers and therapeutic strategies. This study investigates the role of C16orf74 as a potential diagnostic and prognostic biomarker in HNSC. Bioinformatics analyses revealed that C16orf74 is significantly overexpressed in HNSC and is associated with advanced disease stages, therapy resistance, and shorter overall and progression-free survival. A prognostic nomogram integrating C16orf74 expression with clinicopathological features demonstrated robust predictive performance. Functional enrichment and immune infiltration analyses suggest that high C16orf74 expression might contribute to an immunosuppressive tumor microenvironment by reducing key immune cell populations, such as B cells, T cells, and natural killer cells, which are critical for anti-tumor immunity. Moreover, C16orf74 expression was inversely associated with immune checkpoint expression and immunotherapy response, highlighting its potential as a predictive biomarker for immune checkpoint blockade (ICB) efficacy. Drug sensitivity analyses identified potential therapeutic agents, including arsenic trioxide, carmustine, vincristine, quercetin, and carboplatin for patients with high C16orf74 expression. These findings highlight the potential of C16orf74 as a biomarker and therapeutic target to improve HNSC management.

## Introduction

Head and neck squamous cell carcinoma (HNSC) is a highly prevalent and aggressive malignancy, accounting for over 90% of head and neck cancers globally [1]. In 2022, there were approximately 594,000 new cases of head and neck cancers globally, accounting for 3.0% of all cancers and 2.7% of cancer deaths. There are significant regional differences in the incidence of these cancers. Oral cancer has a high incidence in regions such as South Asia, etc., laryngeal cancer is common in regions like Eastern Europe, etc., and nasopharyngeal cancer is prevalent in regions including Southeast Asia, etc. Moreover, the incidence rate among men is about 2–3 times that among women. Despite the use of multidisciplinary treatment strategies, which include surgery, radiotherapy, chemotherapy, and immunotherapy, the prognosis for HNSC patients remains poor [2]. The etiology of HNSC is highly intricate, involving genetic, epigenetic, and environmental factors that collectively contribute to its complexity, particularly in terms of early diagnosis and effective treatment [3]. Therefore, identifying novel diagnostic and prognostic biomarkers is essential for enhancing patient outcomes. HNSC, as an immunosuppressive tumor, demonstrates immune evasion and disrupted immune signaling, both of which are critical to its progression [4]. These immunological characteristics not only exacerbate disease progression but also underscore the potential of immunotherapy as a promising therapeutic approach [5,6]. Nonetheless, resistance to cancer therapies continues to pose a major challenge in clinical management. Additionally, the initiation, progression, and therapy resistance of HNSC are regulated by complex networks of molecular and cellular pathways. While significant strides have been made in recent years to elucidate these pathways, the exact molecular mechanisms driving its tumorigenesis, progression, and drug resistance remain poorly understood. This knowledge gap highlights the necessity for further research to identify new therapeutic targets and ultimately enhance clinical outcomes for HNSC patients.

The C16orf74 locus is located on chromosome 16q24.1, but its precise function remains unclear. Evidence from multiple genome-wide studies suggests that C16orf74 plays an important role in the inflammatory process by regulating tumor necrosis factor (TNF)-α, a key regulatory factor in the inflammatory cascade of chronic inflammatory diseases [7]. Additionally, research indicates that C16orf74 is a hypoxia-regulated gene involved in modulating the tumor microenvironment [8]. Furthermore, studies have shown that C16orf74 expression correlates with potential prognostic factors in various cancer types [9–13]. In head and neck cancer, Winter et al. demonstrated that the median RNA expression level of C16orf74 functions as an independent prognostic marker for recurrence-free survival [8]. Moreover, C16orf74 expression is significantly upregulated in lymph node-positive metastases of patients with oral squamous cell carcinoma of the tongue [14]. However, the specific role and underlying mechanisms of C16orf74 in HNSC remain poorly understood. While its significance has been investigated in other cancer types, limited knowledge exists regarding its function in HNSC. This gap highlights the need for further investigation. A comprehensive analysis of the expression levels, clinical relevance, and molecular mechanisms of C16orf74 in HNSC is essential to uncover new insights and develop targeted therapeutic strategies for this disease.

In this study, we initially investigated the expression of C16orf74 and its clinical significance in HNSC. We subsequently developed a novel nomogram integrating clinicopathological features with C16orf74 expression to enhance the accuracy of patient prognosis predictions. To uncover potential mechanisms underlying the role of C16orf74 in HNSC, we performed weighted correlation network analysis (WGCNA), gene ontology (GO) analysis, and gene set enrichment analysis (GSEA). By combining these approaches, this study aims to elucidate the role of C16orf74 in HNSC and contribute to the development of more effective therapeutic strategies.

## Methods

### Datasets and processing

Gene expression profiles, somatic mutation data, and clinical information for HNSC patients were retrieved from The Cancer Genome Atlas (TCGA, https://portal.gdc.cancer.gov/) database. Additionally, datasets including GSE23558, GSE30748, GSE31056, GSE184616, GSE42743, and GSE145281 (IMvigor210 immunotherapy cohort) were obtained from the Gene Expression Omnibus (GEO, https://www.ncbi.nlm.nih.gov/geo/) database. All datasets underwent standardized preprocessing, including normalization and log2 transformation, using appropriate R packages to ensure comparability and data quality.

### Differential expression analysis of C16orf74

Pan-cancer differential expression analysis of C16orf74 mRNA across 33 cancer types was performed using the R package TCGAplot [15]. For HNSC-specific analyses, the R package ggplot2 [16] was employed to compare C16orf74 expression between normal and tumor tissues.

### Clinical correlation, diagnostic, and prognostic analysis

The relationship between C16orf74 expression and various clinical characteristics was analyzed using the R package ggplot2. Diagnostic performance was evaluated by receiver operating characteristic (ROC) curve analysis, and the area under the curve (AUC) was calculated using the R package pROC [17]. To assess the prognostic value, univariate Cox regression analysis was conducted using TCGAplot, while survival analyses were performed using the R packages survival (https://CRAN.R-project.org/package=survival) and survminer (https://CRAN.R-project.org/package=survminer).

### Genomic alteration and mutational burden analysis

The genomic alterations of C16orf74, including mutation frequencies, amplifications, and deletions, were analyzed using the cBioPortal Cancer Type Summary module (https://www.cbioportal.org/). The R maftools [18], ggplot2, and forestPlot (https://CRAN.R-project.org/package=forestplot) packages were used to assess tumor mutational burden (TMB) and mutation levels between high and low C16orf74 expression groups.

### Nomogram construction and validation

A prognostic nomogram was constructed to predict HNSC patient outcomes by integrating clinical parameters (age, clinical stage, lymphovascular invasion, perineural invasion) with C16orf74 expression. The R package rms (https://CRAN.R-project.org/package=rms) was used to derive calibration curves and examine the agreement between predicted probabilities and observed outcomes. Predictive performance was further quantified by calculating the concordance index (C-index) and AUC using riskRegression (https://CRAN.R-project.org/package=riskRegression) and timeROC [19] packages.

### WGCNA and functional enrichment analysis

A weighted gene co-expression network was constructed to identify gene modules associated with C16orf74 expression in the TCGA dataset using the R package WGCNA [20]. To ensure a scale-free network, a soft threshold of 9 was applied. Genes with similar expression patterns were grouped into modules, and the module most strongly correlated with C16orf74 was selected for further analysis. Functional annotation and pathway enrichment analysis of the this module were conducted using the R packages clusterProfiler [21] and org.Hs.e.g.,db (https://bioconductor.org/packages/org.Hs.eg.db/).

### GSEA

GSEA was performed to identify enriched Hallmark pathways between high and low C16orf74 expression groups. HNSC patients were stratified based on the median C16orf74 mRNA expression level in the TCGA dataset. The analysis was conducted using the R package clusterProfiler, and pathways with normalized enrichment scores (NES) and adjusted p-values < 0.05 were considered significantly enriched.

### Immune infiltration analysis

To evaluate immune infiltration and the tumor immune microenvironment, the immune score, stromal score, and ESTIMATE score were calculated using the R package ESTIMATE [22]. Single-sample gene set enrichment analysis (ssGSEA) was performed using the GSVA [23] package to assess the infiltration levels of 28 tumor-infiltrating immune cells. Correlations between immune cell infiltration and C16orf74 expression were evaluated using Spearman correlation analysis.

### Drug sensitivity analysis

The relationship between C16orf74 expression and drug sensitivity was assessed using the NCI-60 cell line panel, which includes 60 tumor cell lines and sensitivity data for 792 chemotherapeutic and targeted agents. Tumor cell lines with more than 60% missing data were excluded. Spearman correlation analysis was performed to determine the association between C16orf74 expression and drug sensitivity.

### Statistical analysis

All statistical analyses were conducted using R software (version 4.4.2). A p-value < 0.05 was considered statistically significant. Statistical significance levels were denoted as follows: *p < 0.05, **p < 0.01, ***p < 0.001, ****p < 0.0001.

## Results

### Elevated expression of C16orf74 in HNSC

**Fig 1:**
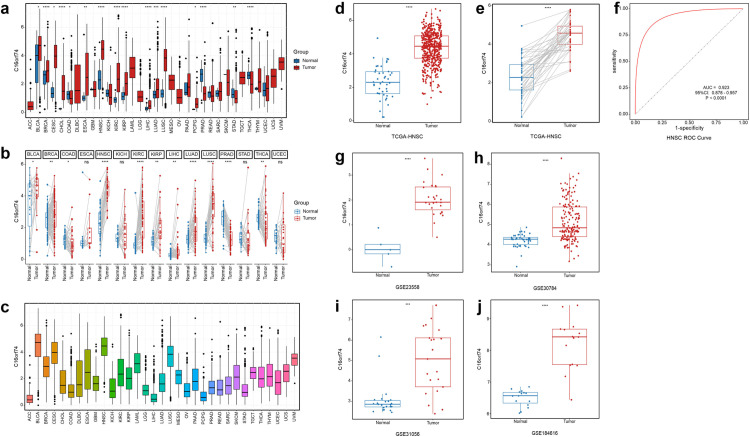
Elevated expression of C16orf74 in HNSC and other cancers. Pan-cancer analysis of C16orf74 expression in normal and tumor tissues from TCGA datasets (a). Paired analysis of C16orf74 expression in tumor and adjacent normal tissues from TCGA datasets (b). Comparative analysis of C16orf74 expression across multiple cancer types (c). Boxplot and paired plot of C16orf74 expression in HNSC tumor and normal tissues from TCGA datasets (d-e). ROC curve analysis of C16orf74 expression in HNSC (f). Independent validation of C16orf74 expression in HNSC using GSE23558, GSE30784, GSE31056, and GSE184616 datasets (g-j). TCGA, The Cancer Genome Atlas; HNSC, head and neck squamous cell carcinoma; ROC, receiver operating characteristic. Statistical significance: *p < 0.05, **p < 0.01, ***p < 0.001, ****p < 0.0001, ns: not significant.

To evaluate C16orf74 expression in human cancers, we analyzed its differential expression between normal and tumor tissues. As illustrated in Fig 1a, C16orf74 expression was found to be significantly upregulated in multiple cancer types, including bladder urothelial carcinoma (BLCA), breast invasive carcinoma (BRCA), cervical squamous cell carcinoma and endocervical adenocarcinoma (CESC), cholangiocarcinoma (CHOL), esophageal carcinoma (ESCA), head and neck squamous cell carcinoma (HNSC), kidney renal clear cell carcinoma (KIRC), kidney renal papillary cell carcinoma (KIRP), liver hepatocellular carcinoma (LIHC), lung adenocarcinoma (LUAD), lung squamous cell carcinoma (LUSC), and pheochromocytoma and paraganglioma (PCPG). A similar increase in C16orf74 expression was observed in paired tumor and paracancerous tissues of BLCA, BRCA, HNSC, KIRC, KIRP, LIHC, LUAD, and LUSC (Fig 1b). Furthermore, comparative analysis revealed that the expression level of C16orf74 was particularly higher in BLCA, HNSC, and CESC compared to other cancer types (Fig 1c). Notably, HNSC exhibited significantly elevated C16orf74 expression (p < 0.0001; Fig 1d–e). ROC curve analysis demonstrated that C16orf74 expression could reliably distinguish HNSC from normal tissues, with an AUC of 0.923 (95% confidence interval: 0.875–0.957), suggesting its potential utility as a diagnostic biomarker for HNSC (Fig 1f). Additionally, these findings were further validated by analyzing multiple independent datasets, including GSE23558, GSE30784, GSE31056, and GSE184616, which consistently corroborated the results derived from the TCGA dataset (Fig 1g–j).

### C16orf74 is associated with adverse clinical characteristics of HNSC

**Fig 2:**
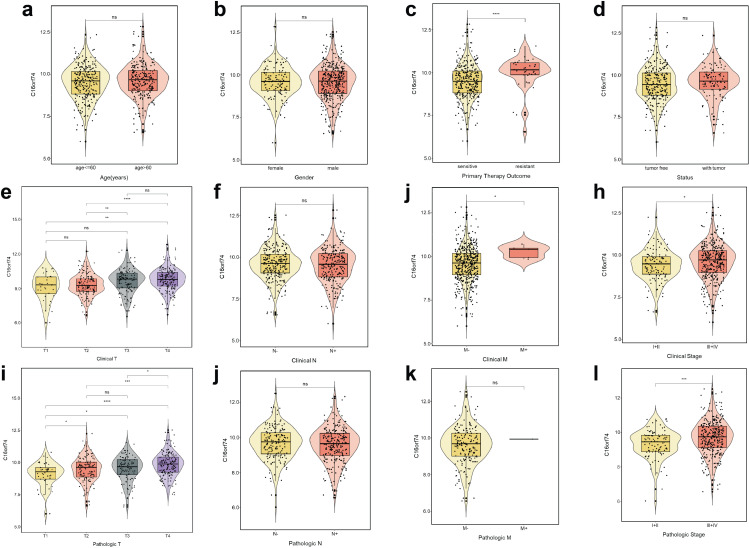
C16orf74 is associated with adverse clinical characteristics of HNSC. Association of C16orf74 expression with age (a), gender (b), primary therapy outcome (c), tumor status (d), clinical T stage (e), clinical N stage (f), clinical M stage (g), clinical stage (h), pathologic T stage (i), pathologic N stage (j), pathologic M stage (k), and pathologic stage (l) based on TCGA database analysis. Statistical significance: *p < 0.05, **p < 0.01, ***p < 0.001, ****p < 0.0001, ns: not significant.

Associations between C16orf74 expression levels and clinical characteristics were analyzed using the TCGA database, which revealed significant correlations between elevated C16orf74 expression and several clinical parameters, including primary therapy outcome, clinical T stage, clinical M stage, clinical stage, pathologic T stage, and pathologic stage (Fig 2a–l). Specifically, patients with HNSC exhibiting resistant primary therapy outcomes, higher clinical and pathologic T stages, and advanced clinical and pathologic stages showed markedly higher C16orf74 expression levels. In addition, subgroup survival analysis was also conducted (S1 Fig). These findings indicate that C16orf74 is associated with adverse clinical features in HNSC and further suggest its potential role as a prognostic biomarker linked to poor outcomes.

### Elevated C16orf74 expression is correlated with poor outcome in HNSC patients

**Fig 3:**
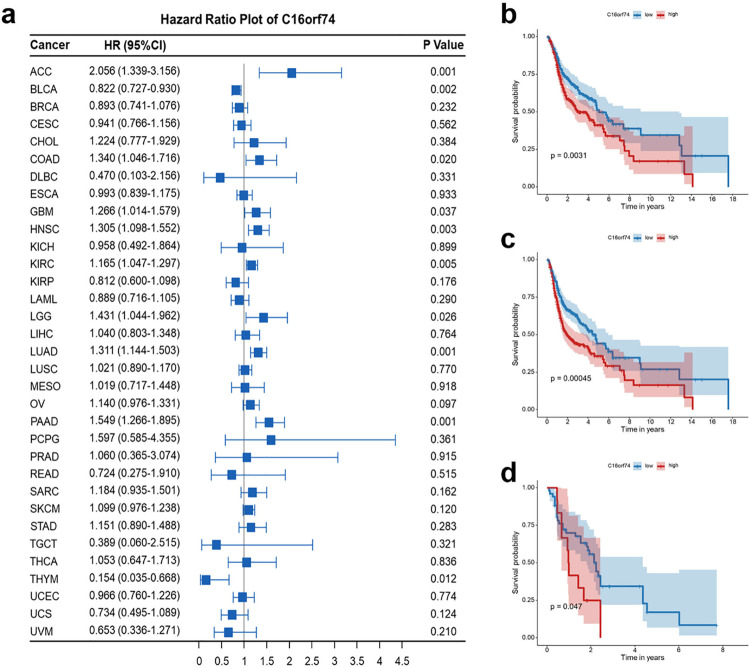
Elevated C16orf74 expression is associated with poor prognosis in HNSC patients. Pan-cancer univariate Cox regression analysis of C16orf74 expression from TCGA datasets (a). Kaplan-Meier survival analysis of OS (b) and PFS (c) in HNSC patients from TCGA datasets. Kaplan-Meier survival analysis of OS in HNSC patients from the GSE42743 dataset (d).

Here, we evaluated the prognostic significance of C16orf74. Specifically, as shown in Fig 3a, C16orf74 was identified as a poor prognostic factor in several cancers, including adrenocortical carcinoma (ACC), colon adenocarcinoma (COAD), glioblastoma (GBM), HNSC, kidney renal clear cell carcinoma (KIRC), lower-grade glioma (LGG), lung adenocarcinoma (LUAD), and pancreatic adenocarcinoma (PAAD). In contrast, it served as a favorable prognostic factor in bladder urothelial carcinoma (BLCA) and thymoma (THYM). Kaplan-Meier survival analysis revealed a significant association between C16orf74 expression levels and prognosis in HNSC patients, with patients exhibiting high C16orf74 expression having shorter OS (Fig 3b) and progression-free survival (PFS) (Fig 3c). Furthermore, an independent analysis of the GEO database (GSE42743) provided additional evidence, confirming that elevated C16orf74 expression is associated with worse OS in HNSC patients (Fig 3d).

### Gene mutation analysis

**Fig 4:**
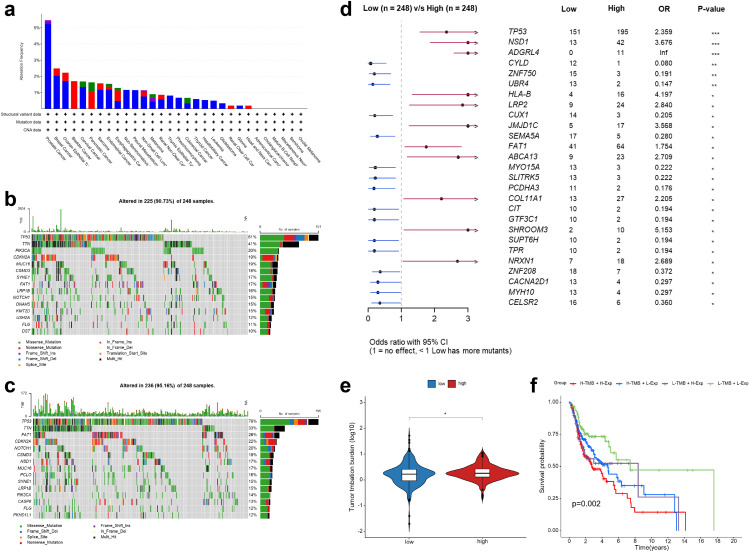
Gene mutation analysis of C16orf74 and its association with TMB in HNSC. Alteration frequency of C16orf74 across various cancer types based on the cBioPortal database (a). Mutation profiles of patients with low (b) and high (c) C16orf74 expression levels in HNSC. Comparison of mutation frequencies for specific genes between low and high C16orf74 expression groups (d). Comparison of TMB between low and high C16orf74 expression groups in HNSC (e). Kaplan-Meier survival analysis of HNSC patients stratified by C16orf74 expression levels and TMB (f). HNSC, head and neck squamous cell carcinoma; TMB, tumor mutation burden. Statistical significance: *p < 0.05, **p < 0.01, ***p < 0.001.

First, an analysis using the cBioPortal database revealed the alteration frequency of C16orf74 across various cancer types (Fig 4a). The findings demonstrated that prostate cancer exhibited the highest alteration frequency of C16orf74, predominantly characterized by deep deletion mutations. However, C16orf74 mutations were nearly absent in head and neck cancer. To further explore the functional implications of these alterations, we subsequently examined the relationship between mutations and C16orf74 expression levels (Fig 4b–c). The analysis revealed that patients with low C16orf74 expression exhibited a significantly lower mutation rate (90.73%) compared to those with high C16orf74 expression (95.16%). The significantly different mutation profiles between high and low C16orf74 expression groups are illustrated in Fig 4d. Genes such as TP53, NSD1, ADGRL4, and HLA-B showed a higher mutation frequency in patients with high C16orf74 expression (p < 0.01). Conversely, CYLD, ZNF750, and UBR4 exhibited more frequent mutations in patients with low C16orf74 expression (p < 0.01). In addition to mutation profiles, we compared the TMB between patients with high and low C16orf74 expression (Fig 4e–f). Notably, HNSC patients with high C16orf74 expression exhibited a significantly high level of TMB (Fig 4e). Kaplan-Meier survival analysis indicated that patients with both high C16orf74 expression and high TMB had the poorest survival outcomes compared to other groups (Fig 4f).

### Constructed nomogram on the basis of C16orf74 predicts patient prognosis

**Fig 5:**
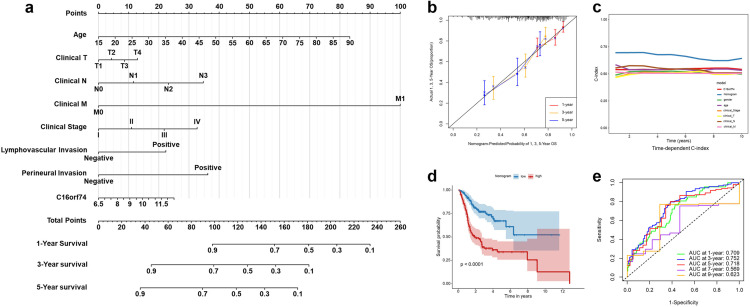
Constructed nomogram based on C16orf74 predicts prognosis in HNSC patients. Nomogram integrating C16orf74 expression and clinicopathological factors for predicting 1-, 3-, and 5-year OS in HNSC patients (a). Calibration curves of the nomogram for 1-, 3-, and 5-year OS (b). Time-dependent C-index comparison between the nomogram and other clinical indicators (c). Kaplan-Meier survival analysis of OS in HNSC patients stratified by nomogram scores (d). ROC curves showing the nomogram’s predictive performance for 1-, 3-, 5-year, 7-year and 9-year OS. (e). HNSC, head and neck squamous cell carcinoma; OS, overall survival; ROC, receiver operating characteristic.

To enhance the accuracy of prognosis prediction for HNSC, we constructed a nomogram model incorporating the expression of C16orf74 along with clinicopathological factors, including age, clinical stage, lymphovascular invasion, and perineural invasion, within the TCGA cohort (Fig 5a). The calibration curves for predicting 1-, 3-, and 5-year OS demonstrated that the nomogram exhibited excellent alignment between predicted and observed outcomes (Fig 5b). The C-index of the nomogram was notably higher compared to the predictive capability of using C16orf74 expression alone (Fig 5c). Furthermore, Kaplan-Meier survival analysis revealed that patients with high nomogram scores exhibited significantly poorer overall survival, as depicted in Fig 5d. The nomogram’s ROC AUC values for predicting 1-, 3-, 5-, 7- and 9-year OS were 0.709, 0.752, 0.718, 0.569 and 0.623 respectively (Fig 5e). These results underscore the robust performance of the C16orf74-based nomogram in reliably predicting the prognosis of HNSC patients.

### WGCNA analysis, functional enrichment analysis and GSEA analysis

**Fig 6:**
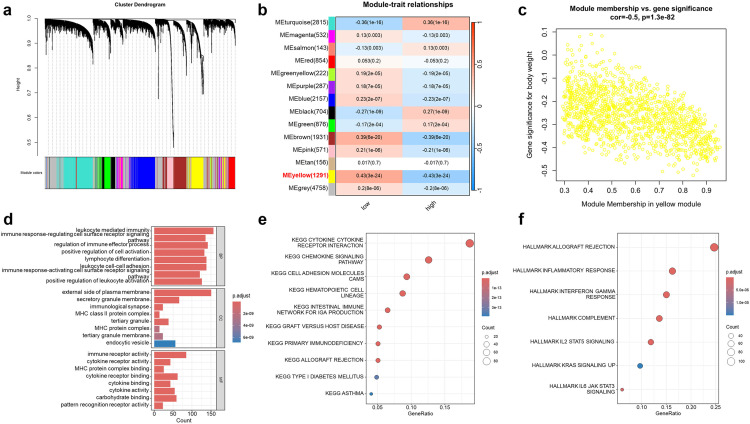
WGCNA and functional enrichment analysis. Clustering dendrogram of genes based on a topological overlap matrix (a). Module-trait relationships showing the correlation between gene modules and C16orf74 expression levels (b). Yellow module genes scatter plots (c). GO (d), KEGG (e) and hallmark pathway (f) enrichment analysis of yellow module genes. WGCNA, weighted gene co-expression network analysis; GO, Gene Ontology; KEGG, Kyoto Encyclopedia of Genes and Genomes.

Using the TCGA dataset, we constructed a WGCNA network to explore the regulatory relationships associated with C16orf74 in HNSC. A soft threshold β of 9 was applied, leading to the identification of 14 gene modules using the topological overlap matrix (TOM) (Fig 6a). These modules were labeled as follows: turquoise (2815 genes), magenta (532), salmon (143), red (854), greenyellow (222), purple (287), blue (2157), black (704), green (876), brown (1931), pink (571), tan (156), yellow (1291), and grey (4758) (Fig 6b). Among these modules, the yellow module was of particular interest due to its strong negative correlation with C16orf74 expression. Subsequent analysis demonstrated that the yellow module exhibited the strongest negative correlation with C16orf74 expression (correlation coefficient = -0.5, p = 1.3e-82) (Fig 6b–c). GO enrichment analysis revealed that genes within the yellow module were predominantly associated with leukocyte-mediated immunity (BP), the external side of the plasma membrane (CC), and immune receptor activity (MF) (Fig 6d). In addition, KEGG enrichment analysis identified the cytokine-cytokine receptor interaction pathway as the most significantly enriched pathway (Fig 6e). These findings collectively suggest that the yellow module may play a critical role in immune regulation. Furthermore, Hallmark pathway enrichment analysis highlighted that these genes were primarily involved in immune-related processes, including allograft rejection, inflammatory response, interferon-gamma response, complement activation, IL2-STAT5 signaling, and IL6-JAK-STAT3 signaling (Fig 6f).

**Fig 7:**
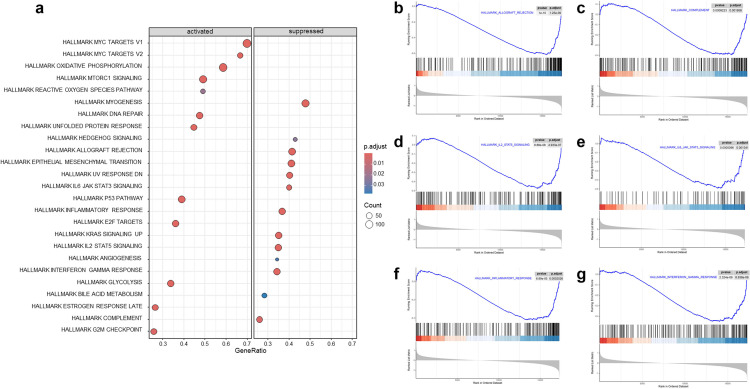
GSEA analysis. Hallmark pathway enrichment analysis of C16orf74-associated signaling pathways in the TCGA dataset (a). GSEA enrichment plots for immune-related pathways, including allograft rejection (b), complement activation (c), IL2-STAT5 signaling (d), IL6-JAK-STAT3 signaling (e), inflammatory response (f), and interferon-gamma response (g). GSEA, gene set enrichment analysis; TCGA, The Cancer Genome Atlas.

GSEA was also performed to further elucidate the biological functions of C16orf74 in HNSC. Hallmark pathway enrichment analysis identified several C16orf74-associated signaling pathways in the TCGA dataset, including MYC targets, oxidative phosphorylation, mTORC1 signaling, DNA repair, myogenesis, interferon response, epithelial-mesenchymal transition, allograft rejection, and inflammatory response, among others (Fig 7a). Interestingly, many of these pathways overlap with those identified in the yellow module, particularly the immune-related pathways. Notably, immune-related pathways, including allograft rejection, inflammatory response, interferon-gamma response, complement activation, and the IL6-JAK-STAT3 signaling pathway, were significantly downregulated in the high C16orf74 expression group (Fig 7b–g). In the KEGG results, there are also immune-related pathways such as the “T_CELL_RECEPTOR_SIGNALING_PATHWAY” and the “B_CELL_RECEPTOR_SIGNALING_PATHWAY”. At the same time, there is also the “JAK_STAT_SIGNALING_PATHWAY” (JAK - STAT signaling pathway), where abnormal activation of this pathway is closely associated with the proliferation, survival, and metastasis of tumor cells (S2A Fig). These GO analysis results reflect tumor - related pathways from multiple aspects. In terms of biological processes, processes such as B - cell proliferation, regulation of B - cell activation, αβT - cell activation, and adaptive immune response are closely related to tumor immune escape and the body’s immune response to tumors. Regarding cellular components, MHC protein complexes are involved in antigen presentation, which is crucial for tumor immune surveillance, while the T - cell receptor complex is related to the recognition of tumor antigens by T cells. In terms of molecular functions, immune receptor activity plays an important role in initiating immune responses against tumors, and cytokine receptor activity and cytokine binding affect the functions of immune cells and the growth of tumor cells in the tumor microenvironment (S2 Fig). This downregulation underscores the potential immunosuppressive role of high C16orf74 expression in HNSC.

### C16orf74 is involved in immune cell infiltration in HNSC

**Fig 8:**
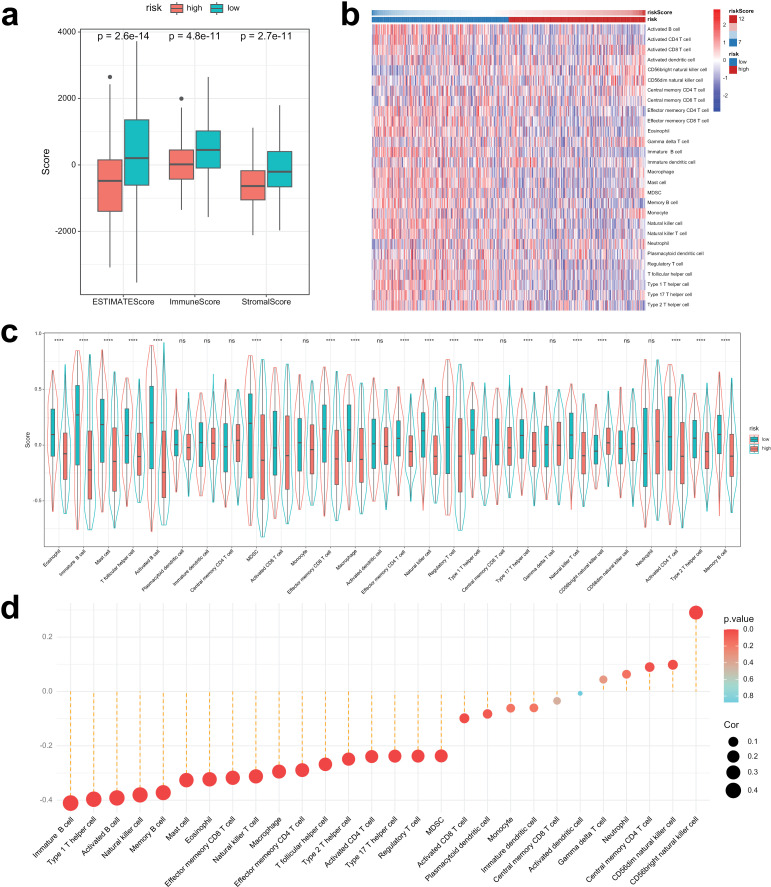
C16orf74 is involved in immune cell infiltration in HNSC. Comparison of ESTIMATE, immune, and stromal scores between low- and high-C16orf74 expression groups in HNSC patients (a). Heatmap of the infiltration of various immune cell types in low- and high-C16orf74 expression groups (b). Boxplots comparing infiltration levels of immune cell types between low- and high-C16orf74 expression groups (c). Correlation analysis between C16orf74 expression and immune cell infiltration (d). HNSC, head and neck squamous cell carcinoma.

A comprehensive analysis was conducted to investigate immune infiltration and the tumor immune microenvironment in HNSC. To begin, we compared the immune score, stromal score, and ESTIMATE score to assess immune status in patients with high versus low C16orf74 expression. As expected, HNSC patients with high C16orf74 expression exhibited significantly lower immune, stromal, and ESTIMATE scores (Fig 8a). To elucidate this further, we conducted ssGSEA analysis to clarify the relationship between C16orf74 and tumor-infiltrating immune cells. The results, as illustrated in Fig 8b, showed that patients with high C16orf74 expression demonstrated significantly lower scores for most immune cells compared to those with low C16orf74 expression. Specifically, the infiltration levels of 18 immune cell types were significantly reduced in the high-C16orf74 expression group. Conversely, only one immune cell type, the CD56bright natural killer cell, exhibited a significant increase in the high-C16orf74 expression group (Fig 8c). Further correlation analysis revealed that C16orf74 expression was inversely associated with most immune cell types (Fig 8d).

**Fig 9:**
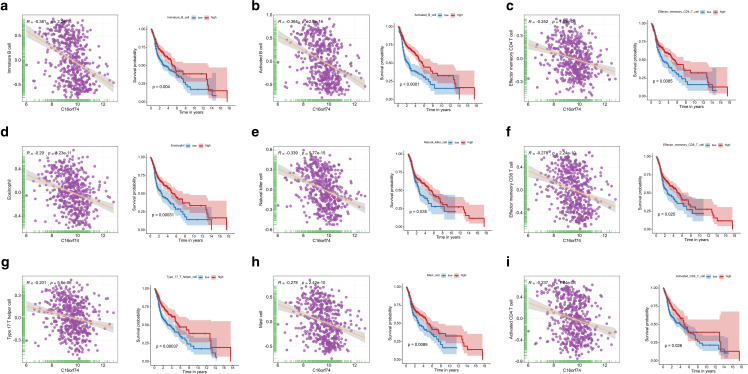
Correlation and survival curves between C16orf74 expression and immune cell infiltration. Immature B cells (a), activated B cells (b), effector memory CD4 T cells (c), eosinophils (d), natural killer cells (e), effector memory CD8 T cells (f), type 17 T helper cells (g), mast cells (h), and activated CD4 T cells (i).

Among immune cells negatively correlated with C16orf74 expression (cor < -0.2, p < 0.001), several were significantly associated with patient prognosis. Notably, Fig 9a–i demonstrates that patients with low infiltration levels of specific immune cells (e.g., immature B cells, activated B cells, effector memory CD4 T cells, eosinophils, natural killer cells, effector memory CD8 T cells, type 17 T helper cells, mast cells, and activated CD4 T cells) exhibited significantly worse prognoses compared to those with higher infiltration levels. These findings underscore that the aforementioned nine immune cell types may play a pivotal role in the poor prognosis linked to high C16orf74 expression in HNSC. Thus, C16orf74 likely contributes to poor prognosis in HNSC patients by impairing their immune status.

### Correlation analysis of C16orf74 expression with immunotherapy response

**Fig 10:**
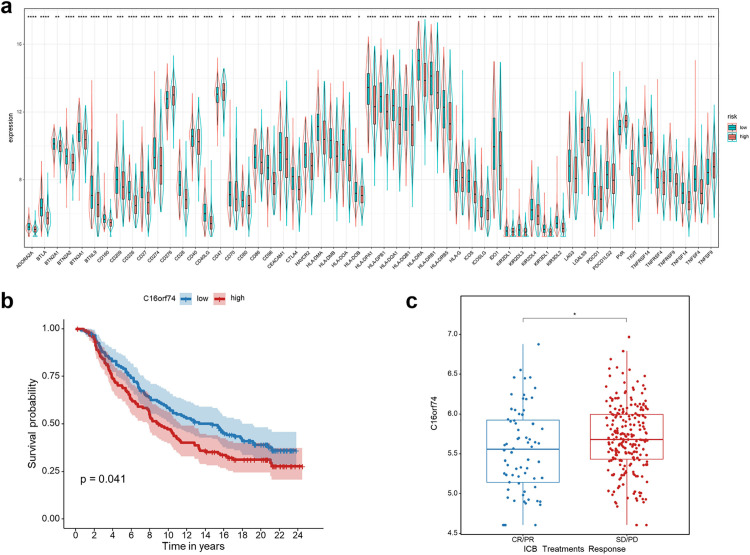
Correlation analysis of C16orf74 expression with immunotherapy response. Comparison of immune checkpoint expression levels between low- and high-C16orf74 expression groups in HNSC patients (a). Kaplan-Meier survival analysis between low- and high-C16orf74 expression groups in the IMvigor210 cohort (b). C16orf74 expression levels between patients with complete or partial response (CR/PR) and those with stable or progressive disease (SD/PD) in the IMvigor210 cohort (c). HNSC, head and neck squamous cell carcinoma; CR, complete response; PR, partial response; SD, stable disease; PD, progressive disease.

Furthermore, we explored the relationship between C16orf74 expression and the effectiveness of immune checkpoint blockade (ICB) treatments in HNSC. Our analysis revealed that the expression of immune checkpoints was significantly higher in the low-C16orf74 expression group (Fig 10a), suggesting that these patients might exhibit an enhanced responsiveness to ICB treatments. To validate these findings, participants in the IMvigor210 immunotherapy cohort (GSE145281) were stratified into high- and low-C16orf74 expression groups. Consistent with our hypothesis, Fig 10b illustrates that, patients in the low-C16orf74 expression group exhibited improved survival outcomes following ICB treatment compared to those in the high-C16orf74 expression group. Moreover, patients who achieved a complete or partial response to ICB treatment were observed to have significantly lower C16orf74 expression levels compared to those with stable or progressive disease (Fig 10c). These results collectively highlight the potential clinical relevance of C16orf74 as a biomarker for predicting ICB treatment outcomes in HNSC.

### Correlation between C16orf74 expression and drug sensitivity

**Fig 11:**
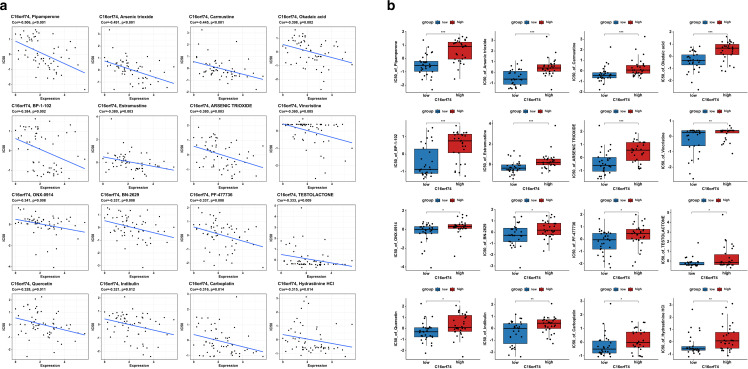
Correlation between C16orf74 expression and drug sensitivity. Scatter plots (a) and boxplots (b) showing the negative correlation between C16orf74 expression and sensitivity to various drugs. Statistical significance: *p < 0.05, **p < 0.01, ***p < 0.001.

To identify potential therapeutic drugs for patients with high C16orf74 expression, we conducted an analysis of the relationship between C16orf74 expression and drug sensitivity. Our analysis revealed a negative correlation between C16orf74 expression and sensitivity to several drugs, including Pipamperone, Arsenic trioxide, Carmustine, Okadaic acid, BP-1–102, Estramustine, Arsenic trioxide, Vincristine, ONX-0914, BN-2629, PF-477736, Testolactone, Quercetin, Indibulin, Carboplatin, and Hydrastinine HCl (Fig 11a–b). This negative correlation implies that high C16orf74 expression may increase the effectiveness of these drugs. These findings suggest that targeting these drugs could offer a promising strategy to improve therapeutic outcomes in patients with elevated C16orf74 expression.

## Discussion

HNSC is one of the most prevalent and aggressive malignancies worldwide [1]. Despite advances in multimodal treatment strategies, including surgery, radiotherapy, chemotherapy, and immunotherapy, the prognosis for HNSC patients remains suboptimal [6]. Surgical resection can be curative for early-stage HNSC; however, patients with advanced-stage disease often face limited treatment efficacy, frequent recurrence, and metastasis, resulting in a dismal 5-year survival rate of less than 50%. These disheartening statistics underscore the urgent need for innovative therapeutic strategies and enhanced prognostic tools to improve patient outcomes. Immunotherapy has emerged as a cornerstone in the treatment of recurrent or metastatic HNSC, particularly as a first-line option for patients with advanced disease [24–27]. Compared to traditional chemotherapy, the combination of immunotherapy and chemotherapy has demonstrated significantly improved clinical outcomes, including prolonged overall survival and enhanced response rates. Furthermore, the latest clinical trial results have expanded the indications for immunotherapy, with growing evidence supporting its integration into earlier stages of HNSC management to improve long-term outcomes [28–31]. Despite these advances, a substantial proportion of HNSC patients fail to benefit from immunotherapy, suggesting that individual differences in the TME may play a pivotal role in determining therapeutic outcomes [4,32]. Thus, a deeper understanding of these heterogeneities is essential for optimizing immunotherapy strategies and broadening its benefits to a larger patient population.

TME is a complex and dynamic ecosystem comprising tumor cells, immune cells, stromal cells, and extracellular matrix components, all of which interact to influence tumor initiation, progression, and metastasis [33]. In HNSC, the TME is characterized by an immunosuppressive milieu that promotes immune evasion and interferes with the efficacy of immunotherapy. For instance, the infiltration of regulatory T cells, myeloid-derived suppressor cells, and tumor-associated macrophages can suppress anti-tumor immune responses, while factors such as hypoxia and extracellular matrix remodeling further exacerbate immune suppression [34–37]. These immunosuppressive mechanisms not only highlight the challenges of overcoming resistance to immunotherapy but also underscore the importance of targeting the TME to enhance treatment efficacy. Advances in high-throughput sequencing and single-cell RNA sequencing technologies have revolutionized the study of the TME, enabling a more comprehensive understanding of its cellular and molecular composition. These technologies provide an unprecedented opportunity to elucidate the intricate interactions between the TME and immunotherapy, thereby paving the way for the identification of novel biomarkers and therapeutic targets. Further research into the TME’s role in shaping treatment responses is critical to overcoming current limitations and achieving more personalized and effective therapeutic strategies for HNSC patients.

This study comprehensively investigated the role of C16orf74 in HNSC using RNA expression data from the TCGA and GEO databases. Our initial analysis revealed that C16orf74 was notably overexpressed in HNSC tumor tissues compared to adjacent normal tissues, suggesting its potential as a diagnostic biomarker for HNSC. Clinical association analysis showed that elevated expression of C16orf74 was significantly associated with unfavorable clinical features, including resistance to primary therapy, higher clinical and pathologic T stages, and advanced disease stages. Gene mutation analysis revealed that patients with high C16orf74 expression exhibited a higher mutation rate compared to those with low C16orf74 expression. Survival analysis further indicated that HNSC patients with high C16orf74 expression had shorter OS and PFS. Notably, these findings are consistent with previous reports linking elevated C16orf74 expression to poorer survival outcomes in other malignancies, such as bladder, pancreatic, and lung cancers [9,11–13].

Currently, research on the biological mechanisms of C16orf74 remains limited, with only a few studies exploring its potential role in promoting pancreatic cancer. In pancreatic ductal adenocarcinoma (PDAC), C16orf74 has been identified as a critical mediator of integrin and calcineurin (CN) signaling pathways, both of which are pivotal for cancer cell proliferation, invasion, and metastasis [11]. Localized beneath the cell membrane, C16orf74 binds directly to integrin αVβ3 in its dimeric form, enabling activation of the ILK/PI3K/Akt/mTOR pathway. This activation promotes phosphorylation of Akt and mTOR, subsequently enhancing the activity of MMP2, a critical enzyme driving cancer cell invasiveness. Furthermore, C16orf74 links integrin signals at the cell membrane to CN in the cytoplasm, amplifying downstream signaling cascades. These interactions collectively accelerate PDAC progression by activating oncogenic pathways, such as Akt/mTOR. Toru Nakamura et al. further demonstrated that C16orf74 enhances cell growth and invasion in PDAC by interacting with PPP3CA, which activates the PPP3CA-NFAT pathway and upregulates oncogenic genes [12]. Despite these insights into C16orf74’s mechanisms in PDAC, its functional role and underlying mechanisms in promoting HNSC progression is still unclear.

To investigate the mechanisms underlying the prognostic differences between HNSC patients with high and low C16orf74 expression, we analyzed biological process differences using WGCNA and GSEA. Both analyses revealed notable enrichment of immune-related pathways. Specifically, pathways such as allograft rejection, inflammatory response, interferon-gamma response, complement activation, and the IL6-JAK-STAT3 signaling pathway were significantly downregulated in the high C16orf74 expression group. This consistent downregulation underscores the potential immunosuppressive role of high C16orf74 expression in HNSC. Therefore, we further examined immune infiltration and the tumor immune microenvironment in HNSC. Consistent with expectations, HNSC patients with high C16orf74 expression showed significantly lower immune, stromal, and ESTIMATE scores. Patients with high C16orf74 expression also exhibited significantly reduced scores for most immune cell populations compared to those with low expression. Moreover, several immune cell types negatively correlated with C16orf74 expression were significantly associated with patient prognosis. Notably, patients with low infiltration of specific immune cells—including immature B cells, activated B cells, effector memory CD4 T cells, eosinophils, natural killer cells, effector memory CD8 T cells, type 17 T helper cells, mast cells, and activated CD4 T cells—had significantly worse prognoses than those with higher infiltration levels. These findings suggest that the aforementioned immune cell types may play a critical role in the poor prognosis associated with high C16orf74 expression in HNSC. Previous research has shown that immature and activated B cells are protective factors for OS in breast cancer patients [38]. Similarly, Wu et al. reported that memory T cells exhibit potent anti-tumor activity and are associated with improved prognosis in colorectal cancer patients [39]. Other studies have also indicated that elevated eosinophil and natural killer cell levels are favorable prognostic factors in cancer patients [40–42]. Taken together, these findings suggest that C16orf74 may contribute to poor prognosis in HNSC by impairing the immune status of patients, particularly through reductions in key immune cell populations with protective roles.

Additionally, the study examined the relationship between C16orf74 expression and the effectiveness of ICB treatments in HNSC. The analysis revealed that patients with low C16orf74 expression who received ICB therapy exhibited significantly improved survival outcomes compared to those with high expression. Patients achieving a complete or partial response to ICB treatment were observed to have significantly lower C16orf74 expression levels compared to those with stable or progressive disease. These observations underscore the clinical relevance of C16orf74 expression as a predictive biomarker for stratifying HNSC patients based on their likelihood of responding to ICB therapy. Lastly, we explored potential therapeutic agents, including arsenic trioxide, carmustine, vincristine, quercetin, and carboplatin, for patients with high C16orf74 expression through correlation analysis between C16orf74 expression and drug sensitivity. This analysis provides a foundation for identifying alternative therapeutic strategies tailored to patients with high C16orf74 expression.

This study also has several limitations. First, our analyses were based on retrospective datasets, which may introduce biases such as batch effects and patient heterogeneity. Prospective validation in independent cohorts is necessary to confirm these findings. Second, functional experiments, such as in vitro and in vivo studies, are essential to elucidate the causal mechanisms of C16orf74 in tumor progression and immune modulation. Future studies integrating proteomics and advanced technologies, such as single-cell sequencing, are needed to address these gaps and further validate C16orf74 as a biomarker and therapeutic target in HNSC.

## Conclusions

This study suggests that C16orf74 could serve as a potential diagnostic and prognostic biomarker in HNSC. C16orf74 is significantly overexpressed in HNSC and is associated with advanced disease stages, therapy resistance, and poor survival outcomes. Mechanistic insights from functional enrichment and immune infiltration analyses indicate that high C16orf74 expression might contribute to an immunosuppressive tumor microenvironment by reducing key immune cell populations, such as B cells, T cells, and natural killer cells. Furthermore, its inverse association with immune checkpoint expression and immunotherapy response suggests its potential as a predictive biomarker for ICB efficacy. Drug sensitivity analyses also identified candidate agents that could serve as alternative therapeutic options for patients with high C16orf74 expression. Future prospective studies and experimental validation are essential to confirm the functional role of C16orf74 in HNSC and its clinical utility as a biomarker and therapeutic target.

## Supporting information

S1 Fig Subgroup survival analysis of C16orf74 in HNSC. (TIF)

S2 Fig KEGG and GO enrichment analysis based on GSEA. (TIF)

S3 File TCGA-DATA. (ZIP)

S4 File GEO-DATA1. (ZIP)

S5 File GEO-DATA2. (ZIP)
