# Supplementary figures and images for: C16orf74 is a novel prognostic biomarker and associates with immune infiltration in head and neck squamous cell carcinoma
Source: PLoS One. 2025 May 7;20(5):e0322701. doi: 10.1371/journal.pone.0322701 (PMC12057912; doi:10.1371/journal.pone.0322701)

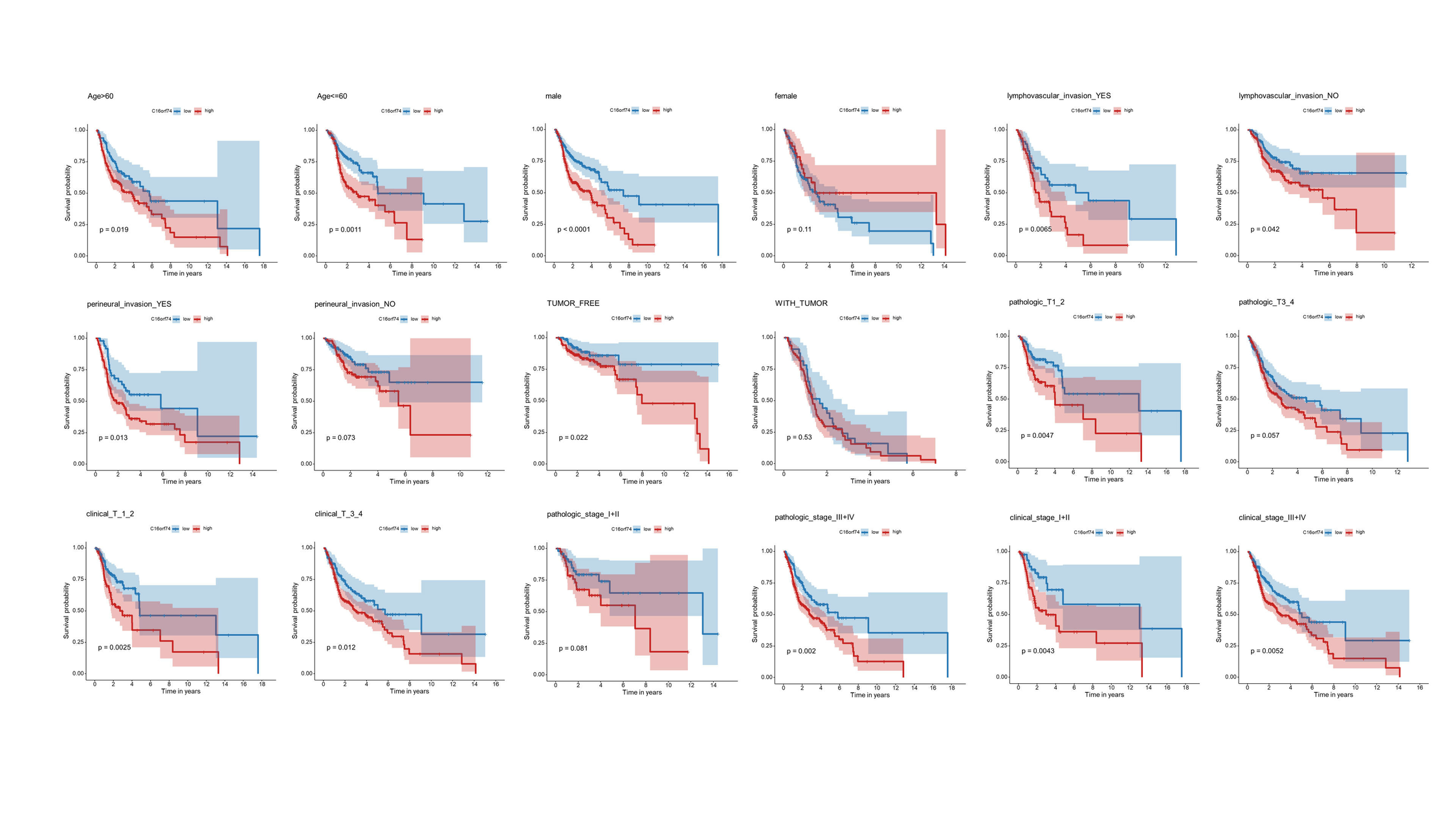

Supplement: S1 Fig — (TIF) [file pone.0322701.s001.tif]

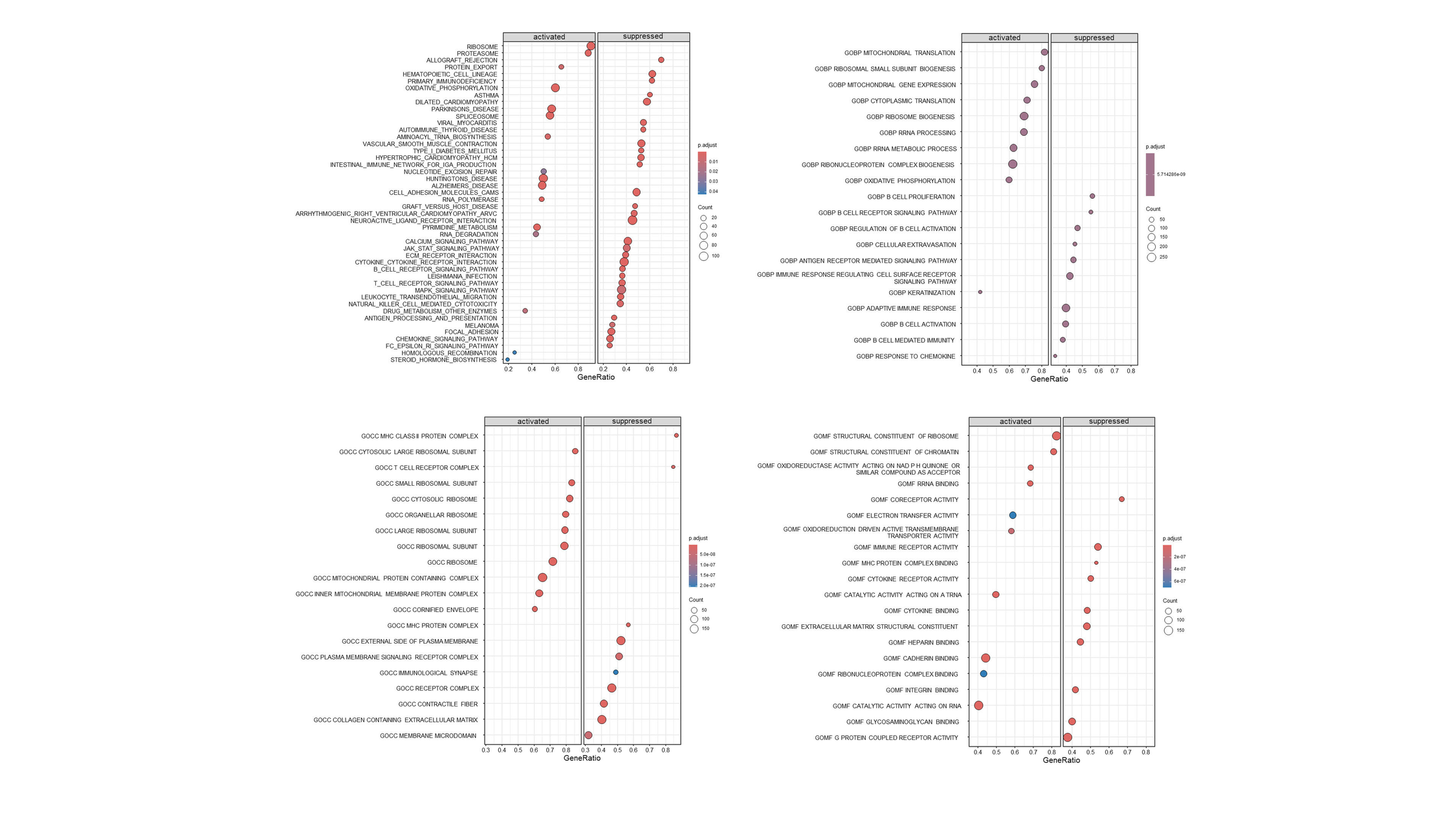

Supplement: S2 Fig — (TIF) [file pone.0322701.s002.tif]
