# Supplementary material for: C16orf74 is a novel prognostic biomarker and associates with immune infiltration in head and neck squamous cell carcinoma
Source: PLoS One. 2025 May 7;20(5):e0322701. doi: 10.1371/journal.pone.0322701 (PMC12057912; doi:10.1371/journal.pone.0322701)
Supplement: S3 File — (ZIP) [file pone.0322701.s003.zip › ALL R scripts.docx]

rm(list = ls())

library(tidyr)

library(dplyr)

library(tibble)

library(export)

# if(!require("BiocManager")) install.packages("BiocManager",update = F,ask = F)

#

# cran_packages=c("magrittr",

# "dplyr",

# "tibble",

# "ggpubr",

# "stringr",

# "reshape2",

# "psych",

# "limma",

# "circlize",

# "grid",

# "fmsb",

# "survival",

# "survminer",

# "forestplot",

# "pROC",

# "tinyarray",

# "ggplot2",

# "patchwork",

# "ggsci",

# "RColorBrewer",

# "pheatmap")

#

# Biocductor_packages=c("edgeR",

# "org.Hs.eg.db",

# "clusterProfiler",

# "enrichplot",

# "ComplexHeatmap",

# "GSVA")

#

# # install packages in CRAN

# for (pkg in cran_packages){

# if (!require(pkg,character.only=T)){

# install.packages(pkg,ask = F,update = F)

# require(pkg,character.only=T)

# }

# }

#

# # install packages in Biocductor

# for (pkg in Biocductor_packages){

# if (!require(pkg,character.only=T)) {

# BiocManager::install(pkg,ask = F,update = F)

# require(pkg,character.only=T)

# }

# }

##

library(TCGAplot)

pan_boxplot("C16orf74",palette=c("#2878b5", "#c82423"),legend="right",method="wilcox.test")

pan_paired_boxplot("C16orf74",palette=c("#2878b5", "#c82423"),legend="right",method="wilcox.test")

pan_tumor_boxplot("C16orf74")

pan_forest("C16orf74",adjust=F)

tcga_boxplot("HNSC","C16orf74",add = "jitter",palette=c("#2878b5", "#c82423"),

legend="none",label="p.signif",method="wilcox.test")

paired_boxplot("HNSC","C16orf74",palette=c("#2878b5", "#c82423"),legend="none",

label="p.signif",method="wilcox.test")

tcga_roc("HNSC","C16orf74")

gene_coexp_heatmap("HNSC","C16orf74",top_n=10, method="pearson")

gene_TMB_radar("C16orf74",method = "pearson")

gene_MSI_radar("C16orf74",method = "pearson")

gene_checkpoint_heatmap("C16orf74",method="pearson",lowcol="blue",highcol="red",cluster_row=T,cluster_col=T,legend=T)

gene_chemokine_heatmap("C16orf74",method="pearson",lowcol="blue",highcol="red",cluster_row=T,cluster_col=T,legend=T)

gene_receptor_heatmap("C16orf74",method="pearson",lowcol="blue",highcol="red",cluster_row=T,cluster_col=T,legend=T)

gene_immustimulator_heatmap("C16orf74",method="pearson",lowcol="blue",highcol="red",cluster_row=T,cluster_col=T,legend=T)

gene_immuinhibitor_heatmap("C16orf74",method="pearson",lowcol="blue",highcol="red",cluster_row=T,cluster_col=T,legend=T)

gene_immucell_heatmap("C16orf74",method="pearson",lowcol="blue",highcol="red",cluster_row=T,cluster_col=T,legend=T)

gene_immunescore_heatmap("C16orf74",method="pearson",lowcol="blue",highcol="red",cluster_row=T,cluster_col=T,legend=T)

gene_immunescore_triangle("C16orf74",method="pearson")

tcga_boxplot("HNSC","C16orf74",add = "jitter",palette="jco",legend="none",label="p.signif",method="wilcox.test")

paired_boxplot("HNSC","C16orf74",palette="jco",legend="none",label="p.signif",method="wilcox.test")

gene_deg_heatmap("HNSC","C16orf74",top_n=20)

gene_gsea_go("HNSC","C16orf74")

gene_gsea_kegg("HNSC","C16orf74")

tcga_roc("HNSC","C16orf74")

gene_gene_scatter(cancer,gene1,gene2,density="F")

gene_methylation_scatter(cancer,gene)

gene_coexp_heatmap("HNSC","C16orf74",top_n=20, method="pearson")

rm(list = ls())

library(tidyr)

library(dplyr)

library(tibble)

library(export)

library(DESeq2)

library(ggpubr)

library(ggplot2)

library(survminer)

library(survival)

library(ggprism)

library(tidyverse)

library(timeROC)

library(data.table)

library(pheatmap)

library(rms)

library(foreign)

library(pec)

load(file = "resource/gene_id_tcga_v36.Rdata")

HNSC <- readRDS("resource/TCGA.rds/TCGA-HNSC.rds")

HNSC <- as.data.frame(HNSC)

HNSC <- HNSC %>%

rownames_to_column("ID")

HNSC <- HNSC %>%

column_to_rownames("ID") %>%

t() %>%

as.data.frame() %>%

mutate(newcolumn = rowMeans(.)) %>%

arrange(desc(newcolumn))

HNSC$sample <- substr(rownames(HNSC),14,15)

HNSC$ID <- substr(rownames(HNSC),1,12)

HNSC <- HNSC %>%

rownames_to_column("TCGA_ID")

HNSC <- HNSC %>%

filter(sample=="01") %>%

dplyr::select(-sample) %>%

dplyr::select(-TCGA_ID) %>%

dplyr::select(-newcolumn) %>%

dplyr::select(ID,everything()) %>%

distinct(ID,.keep_all = T)

HNSC <- HNSC %>%

column_to_rownames("ID") %>%

t() %>%

as.data.frame() %>%

rownames_to_column("gene_id")

expression <- merge(gene_id,HNSC,by="gene_id")

expression <- expression %>%

filter(gene_type=="protein_coding") %>%

dplyr::select(-gene_type) %>%

dplyr::select(-gene_id) %>%

mutate(newcolumn = rowMeans(.[,-1])) %>%

arrange(desc(newcolumn)) %>%

distinct(gene_name,.keep_all = T) %>%

dplyr::select(-newcolumn) %>%

column_to_rownames("gene_name")

exptDesign_TCGA <- data.frame(

condition = rep("tumor", length(colnames(expression))),

row.names = colnames(expression)

)

dds <- DESeqDataSetFromMatrix(

countData = expression,

colData = exptDesign_TCGA,

design = ~ 1)

nrow(dds)

rownames(dds)

dds <- dds[rowMedians(counts(dds))>0,]

nrow(dds)

system.time(vsd <- vst(dds, blind = FALSE))

exprSet_vst <- as.data.frame(assay(vsd))

exprSet_vst <- exprSet_vst %>%

rownames_to_column("ID")

save(exprSet_vst,file = "output/exprSet_vst.Rdata")

rm(list = ls())

load(file = "output/exprSet_vst.Rdata")

exprSet_vst <- exprSet_vst %>%

column_to_rownames("ID") %>%

t() %>%

as.data.frame() %>%

rownames_to_column("TCGA_ID") %>%

dplyr::select(TCGA_ID,C16orf74)

cli=read.table("resource/HNSC_clinical.txt",header=T,sep="\t",check.names=F,row.names=1)

sur=read.table("resource/HNSC_survival.txt",header=T,sep="\t",check.names=F,row.names=1)

colnames(sur)[2] <- "fustat"

colnames(sur)[1] <- "futime"

clinical_new <- cli %>%

rownames_to_column("TCGA_ID")

survival_new <- sur %>%

rownames_to_column("TCGA_ID")

clinical_final <- merge(clinical_new,survival_new,by="TCGA_ID")

clinical_final <- merge(clinical_final,exprSet_vst,by="TCGA_ID")

clinical_final$age <- ifelse(clinical_final$age > 60, ">60", "<=60")

clinical <- clinical_final %>%

dplyr::select(TCGA_ID,fustat,age,gender,histologic_grade,

lymphovascular_invasion,perineural_invasion,cancer_status,primary_therapy_outcome,

pathologic_t,pathologic_n,pathologic_m,pathologic_stage,

clinical_t,clinical_n,clinical_m,clinical_stage,

C16orf74) %>%

column_to_rownames("TCGA_ID")

rt <- clinical %>%

dplyr::select(C16orf74,everything())

age <- rt[,c(1,3)]

age$age <- gsub("<=60", "age<=60", age$age)

age$age <- gsub(">60", "age>60", age$age)

age <- na.omit(age)

age_less_60 <- age[age$age == "age<=60",]

age_more_60 <- age[age$age == "age>60",]

age <- rbind(age_less_60,age_more_60)

colnames(age) <- c("C16orf74","clinical")

age$clinical <- factor(age$clinical, levels = c("age<=60", "age>60"))

comparisons <- list(c("age<=60", "age>60"))

e <- ggplot(age, aes(x = clinical, y = C16orf74))

e + geom_boxplot()

e + geom_violin(trim = FALSE) +

stat_summary(

fun.data = "mean_sdl", fun.args = list(mult = 1),

geom = "pointrange", color = "black"

)

e + geom_violin(aes(fill = clinical), trim = FALSE) +

geom_boxplot(width = 0.5, fill = c("#EED67B", "#EC8874"), color = "black", alpha = 1)+

geom_jitter(shape = 16, position = position_jitter(0.2), color = "black", size = 1) +

scale_fill_manual(values = c("#F7EEC8", "#F7CCBE"))+

theme(legend.position = "none")+

stat_compare_means(comparisons = comparisons,method = "wilcox.test")

fustat <- rt[,c(1,2)]

fustat$fustat <- gsub("0", "Alive", fustat$fustat)

fustat$fustat <- gsub("1", "Dead", fustat$fustat)

fustat <- na.omit(fustat)

Alive <- fustat[fustat$fustat == "Alive",]

Dead <- fustat[fustat$fustat == "Dead",]

fustat <- rbind(Alive,Dead)

colnames(fustat) <- c("C16orf74","clinical")

fustat$clinical <- factor(fustat$clinical, levels = c("Alive", "Dead"))

comparisons <- list(c("Alive", "Dead"))

e <- ggplot(fustat, aes(x = clinical, y = C16orf74))

e + geom_boxplot()

e + geom_violin(trim = FALSE) +

stat_summary(

fun.data = "mean_sdl", fun.args = list(mult = 1),

geom = "pointrange", color = "black"

)

e + geom_violin(aes(fill = clinical), trim = FALSE) +

geom_boxplot(width = 0.5, fill = c("#EED67B", "#EC8874"), color = "black", alpha = 1)+

geom_jitter(shape = 16, position = position_jitter(0.2), color = "black", size = 1) +

scale_fill_manual(values = c("#F7EEC8", "#F7CCBE"))+

theme(legend.position = "none")+

stat_compare_means(comparisons = comparisons,method = "wilcox.test")

gender <- rt[,c(1,4)]

gender <- na.omit(gender)

female <- gender[gender$gender == "female",]

male <- gender[gender$gender == "male",]

gender <- rbind(female,male)

colnames(gender) <- c("C16orf74","clinical")

gender$clinical <- factor(gender$clinical, levels = c("female", "male"))

comparisons <- list(c("female", "male"))

e <- ggplot(gender, aes(x = clinical, y = C16orf74))

e + geom_boxplot()

e + geom_violin(trim = FALSE) +

stat_summary(

fun.data = "mean_sdl", fun.args = list(mult = 1),

geom = "pointrange", color = "black"

)

e + geom_violin(aes(fill = clinical), trim = FALSE) +

geom_boxplot(width = 0.5, fill = c("#EED67B", "#EC8874"), color = "black", alpha = 1)+

geom_jitter(shape = 16, position = position_jitter(0.2), color = "black", size = 1) +

scale_fill_manual(values = c("#F7EEC8", "#F7CCBE"))+

theme(legend.position = "none")+

stat_compare_means(comparisons = comparisons,method = "wilcox.test")

lymphovascular_invasion <- rt[,c(1,6)]

lymphovascular_invasion <- na.omit(lymphovascular_invasion)

NO <- lymphovascular_invasion[lymphovascular_invasion$lymphovascular_invasion == "NO",]

YES <- lymphovascular_invasion[lymphovascular_invasion$lymphovascular_invasion == "YES",]

lymphovascular_invasion <- rbind(NO,YES)

colnames(lymphovascular_invasion) <- c("C16orf74","clinical")

lymphovascular_invasion$clinical <- factor(lymphovascular_invasion$clinical, levels = c("NO", "YES"))

comparisons <- list(c("NO", "YES"))

e <- ggplot(lymphovascular_invasion, aes(x = clinical, y = C16orf74))

e + geom_boxplot()

e + geom_violin(trim = FALSE) +

stat_summary(

fun.data = "mean_sdl", fun.args = list(mult = 1),

geom = "pointrange", color = "black"

)

e + geom_violin(aes(fill = clinical), trim = FALSE) +

geom_boxplot(width = 0.5, fill = c("#EED67B", "#EC8874"), color = "black", alpha = 1)+

geom_jitter(shape = 16, position = position_jitter(0.2), color = "black", size = 1) +

scale_fill_manual(values = c("#F7EEC8", "#F7CCBE"))+

theme(legend.position = "none")+

stat_compare_means(comparisons = comparisons,method = "wilcox.test")

perineural_invasion <- rt[,c(1,7)]

perineural_invasion <- na.omit(perineural_invasion)

NO <- perineural_invasion[perineural_invasion$perineural_invasion == "NO",]

YES <- perineural_invasion[perineural_invasion$perineural_invasion == "YES",]

perineural_invasion <- rbind(NO,YES)

colnames(perineural_invasion) <- c("C16orf74","clinical")

perineural_invasion$clinical <- factor(perineural_invasion$clinical, levels = c("NO", "YES"))

comparisons <- list(c("NO", "YES"))

e <- ggplot(perineural_invasion, aes(x = clinical, y = C16orf74))

e + geom_boxplot()

e + geom_violin(trim = FALSE) +

stat_summary(

fun.data = "mean_sdl", fun.args = list(mult = 1),

geom = "pointrange", color = "black"

)

e + geom_violin(aes(fill = clinical), trim = FALSE) +

geom_boxplot(width = 0.5, fill = c("#EED67B", "#EC8874"), color = "black", alpha = 1)+

geom_jitter(shape = 16, position = position_jitter(0.2), color = "black", size = 1) +

scale_fill_manual(values = c("#F7EEC8", "#F7CCBE"))+

theme(legend.position = "none")+

stat_compare_means(comparisons = comparisons,method = "wilcox.test")

cancer_status <- rt[,c(1,8)]

cancer_status <- na.omit(cancer_status)

TUMOR_FREE <- cancer_status[cancer_status$cancer_status == "TUMOR FREE",]

WITH_TUMOR <- cancer_status[cancer_status$cancer_status == "WITH TUMOR",]

cancer_status <- rbind(TUMOR_FREE,WITH_TUMOR)

colnames(cancer_status) <- c("C16orf74","clinical")

cancer_status$clinical <- factor(cancer_status$clinical, levels = c("TUMOR FREE", "WITH TUMOR"))

comparisons <- list(c("TUMOR FREE", "WITH TUMOR"))

e <- ggplot(cancer_status, aes(x = clinical, y = C16orf74))

e + geom_boxplot()

e + geom_violin(trim = FALSE) +

stat_summary(

fun.data = "mean_sdl", fun.args = list(mult = 1),

geom = "pointrange", color = "black"

)

e + geom_violin(aes(fill = clinical), trim = FALSE) +

geom_boxplot(width = 0.5, fill = c("#EED67B", "#EC8874"), color = "black", alpha = 1)+

geom_jitter(shape = 16, position = position_jitter(0.2), color = "black", size = 1) +

scale_fill_manual(values = c("#F7EEC8", "#F7CCBE"))+

theme(legend.position = "none")+

stat_compare_means(comparisons = comparisons,method = "wilcox.test")

primary_therapy_outcome <- rt[,c(1,9)]

primary_therapy_outcome <- na.omit(primary_therapy_outcome)

Resistant <- primary_therapy_outcome[primary_therapy_outcome$primary_therapy_outcome == "Resistant",]

Sensitive <- primary_therapy_outcome[primary_therapy_outcome$primary_therapy_outcome == "Sensitive",]

primary_therapy_outcome <- rbind(Resistant,Sensitive)

colnames(primary_therapy_outcome) <- c("C16orf74","clinical")

primary_therapy_outcome$clinical <- factor(primary_therapy_outcome$clinical, levels = c("Sensitive", "Resistant"))

comparisons <- list(c("Sensitive", "Resistant"))

e <- ggplot(primary_therapy_outcome, aes(x = clinical, y = C16orf74))

e + geom_boxplot()

e + geom_violin(trim = FALSE) +

stat_summary(

fun.data = "mean_sdl", fun.args = list(mult = 1),

geom = "pointrange", color = "black"

)

e + geom_violin(aes(fill = clinical), trim = FALSE) +

geom_boxplot(width = 0.5, fill = c("#EED67B", "#EC8874"), color = "black", alpha = 1)+

geom_jitter(shape = 16, position = position_jitter(0.2), color = "black", size = 1) +

scale_fill_manual(values = c("#F7EEC8", "#F7CCBE"))+

theme(legend.position = "none")+

stat_compare_means(comparisons = comparisons,method = "wilcox.test")

pathologic_t <- rt[,c(1,10)]

pathologic_t$pathologic_t <- gsub("T1", "I", pathologic_t$pathologic_t)

pathologic_t$pathologic_t <- gsub("T2", "II", pathologic_t$pathologic_t)

pathologic_t$pathologic_t <- gsub("T3", "III", pathologic_t$pathologic_t)

pathologic_t$pathologic_t <- gsub("T4", "IV", pathologic_t$pathologic_t)

pathologic_t <- na.omit(pathologic_t)

I <- pathologic_t[pathologic_t$pathologic_t == "I",]

II <- pathologic_t[pathologic_t$pathologic_t == "II",]

III <- pathologic_t[pathologic_t$pathologic_t == "III",]

IV <- pathologic_t[pathologic_t$pathologic_t == "IV",]

pathologic_t <- rbind(I,II,III,IV)

colnames(pathologic_t) <- c("C16orf74","clinical")

pathologic_t$clinical <- factor(pathologic_t$clinical, levels = c("I","II","III","IV"))

comparisons <- list(c("I","II"), c("I","III"), c("I","IV"), c("II","III"), c("II","IV"), c("III", "IV"))

e <- ggplot(pathologic_t, aes(x = clinical, y = C16orf74))

e + geom_boxplot()

e + geom_violin(trim = FALSE) +

stat_summary(

fun.data = "mean_sdl", fun.args = list(mult = 1),

geom = "pointrange", color = "black"

)

e + geom_violin(aes(fill = clinical), trim = FALSE) +

geom_boxplot(width = 0.5, fill = c("#EED67B", "#EC8874", "#6E7C7D", "#9D84BE"), color = "black", alpha = 1)+

scale_fill_manual(values = c("#F7EEC8", "#F7CCBE", "#BABEBF", "#D1C7E2"))+

geom_jitter(shape = 16, position = position_jitter(0.2), color = "black", size = 1) +

theme(legend.position = "none")+

stat_compare_means(comparisons = comparisons,method = "wilcox.test")

clinical_t <- rt[,c(1,14)]

clinical_t$clinical_t <- gsub("T1", "I", clinical_t$clinical_t)

clinical_t$clinical_t <- gsub("T2", "II", clinical_t$clinical_t)

clinical_t$clinical_t <- gsub("T3", "III", clinical_t$clinical_t)

clinical_t$clinical_t <- gsub("T4", "IV", clinical_t$clinical_t)

clinical_t <- na.omit(clinical_t)

I <- clinical_t[clinical_t$clinical_t == "I",]

II <- clinical_t[clinical_t$clinical_t == "II",]

III <- clinical_t[clinical_t$clinical_t == "III",]

IV <- clinical_t[clinical_t$clinical_t == "IV",]

clinical_t <- rbind(I,II,III,IV)

colnames(clinical_t) <- c("C16orf74","clinical")

clinical_t$clinical <- factor(clinical_t$clinical, levels = c("I","II","III","IV"))

comparisons <- list(c("I","II"), c("I","III"), c("I","IV"), c("II","III"), c("II","IV"), c("III", "IV"))

e <- ggplot(clinical_t, aes(x = clinical, y = C16orf74))

e + geom_boxplot()

e + geom_violin(trim = FALSE) +

stat_summary(

fun.data = "mean_sdl", fun.args = list(mult = 1),

geom = "pointrange", color = "black"

)

e + geom_violin(aes(fill = clinical), trim = FALSE) +

geom_boxplot(width = 0.5, fill = c("#EED67B", "#EC8874", "#6E7C7D", "#9D84BE"), color = "black", alpha = 1)+

scale_fill_manual(values = c("#F7EEC8", "#F7CCBE", "#BABEBF", "#D1C7E2"))+

geom_jitter(shape = 16, position = position_jitter(0.2), color = "black", size = 1) +

theme(legend.position = "none")+

stat_compare_means(comparisons = comparisons,method = "wilcox.test")

pathologic_stage <- rt[,c(1,13)]

pathologic_stage$pathologic_stage <- gsub(" ", "", pathologic_stage$pathologic_stage)

pathologic_stage$pathologic_stage <- gsub("IV", "4", pathologic_stage$pathologic_stage)

pathologic_stage$pathologic_stage <- gsub("III", "3", pathologic_stage$pathologic_stage)

pathologic_stage$pathologic_stage <- gsub("II", "2", pathologic_stage$pathologic_stage)

pathologic_stage$pathologic_stage <- gsub("I", "1", pathologic_stage$pathologic_stage)

pathologic_stage$pathologic_stage <- gsub("1", "I+II", pathologic_stage$pathologic_stage)

pathologic_stage$pathologic_stage <- gsub("2", "I+II", pathologic_stage$pathologic_stage)

pathologic_stage$pathologic_stage <- gsub("3", "III+IV", pathologic_stage$pathologic_stage)

pathologic_stage$pathologic_stage <- gsub("4", "III+IV", pathologic_stage$pathologic_stage)

I_II <- pathologic_stage[pathologic_stage$pathologic_stage == "I+II",]

III_IV <- pathologic_stage[pathologic_stage$pathologic_stage == "III+IV",]

pathologic_stage <- rbind(I_II,III_IV)

colnames(pathologic_stage) <- c("C16orf74","clinical")

pathologic_stage$clinical <- factor(pathologic_stage$clinical, levels = c("I+II","III+IV"))

comparisons <- list(c("I+II", "III+IV"))

e <- ggplot(pathologic_stage, aes(x = clinical, y = C16orf74))

e + geom_boxplot()

e + geom_violin(trim = FALSE) +

stat_summary(

fun.data = "mean_sdl", fun.args = list(mult = 1),

geom = "pointrange", color = "black"

)

e + geom_violin(aes(fill = clinical), trim = FALSE) +

geom_boxplot(width = 0.5, fill = c("#EED67B", "#EC8874"), color = "black", alpha = 1)+

geom_jitter(shape = 16, position = position_jitter(0.2), color = "black", size = 1) +

scale_fill_manual(values = c("#F7EEC8", "#F7CCBE"))+

theme(legend.position = "none")+

stat_compare_means(comparisons = comparisons,method = "wilcox.test")

clinical_stage <- rt[,c(1,17)]

clinical_stage$clinical_stage <- gsub(" ", "", clinical_stage$clinical_stage)

clinical_stage$clinical_stage <- gsub("IV", "4", clinical_stage$clinical_stage)

clinical_stage$clinical_stage <- gsub("III", "3", clinical_stage$clinical_stage)

clinical_stage$clinical_stage <- gsub("II", "2", clinical_stage$clinical_stage)

clinical_stage$clinical_stage <- gsub("I", "1", clinical_stage$clinical_stage)

clinical_stage$clinical_stage <- gsub("1", "I+II", clinical_stage$clinical_stage)

clinical_stage$clinical_stage <- gsub("2", "I+II", clinical_stage$clinical_stage)

clinical_stage$clinical_stage <- gsub("3", "III+IV", clinical_stage$clinical_stage)

clinical_stage$clinical_stage <- gsub("4", "III+IV", clinical_stage$clinical_stage)

I_II <- clinical_stage[clinical_stage$clinical_stage == "I+II",]

III_IV <- clinical_stage[clinical_stage$clinical_stage == "III+IV",]

clinical_stage <- rbind(I_II,III_IV)

colnames(clinical_stage) <- c("C16orf74","clinical")

clinical_stage$clinical <- factor(clinical_stage$clinical, levels = c("I+II","III+IV"))

comparisons <- list(c("I+II", "III+IV"))

e <- ggplot(clinical_stage, aes(x = clinical, y = C16orf74))

e + geom_boxplot()

e + geom_violin(trim = FALSE) +

stat_summary(

fun.data = "mean_sdl", fun.args = list(mult = 1),

geom = "pointrange", color = "black"

)

e + geom_violin(aes(fill = clinical), trim = FALSE) +

geom_boxplot(width = 0.5, fill = c("#EED67B", "#EC8874"), color = "black", alpha = 1)+

geom_jitter(shape = 16, position = position_jitter(0.2), color = "black", size = 1) +

scale_fill_manual(values = c("#F7EEC8", "#F7CCBE"))+

theme(legend.position = "none")+

stat_compare_means(comparisons = comparisons,method = "wilcox.test")

pathologic_n <- rt[,c(1,11)]

pathologic_n$pathologic_n <- gsub("N0", "N-", pathologic_n$pathologic_n)

pathologic_n$pathologic_n <- gsub("N1", "N+", pathologic_n$pathologic_n)

pathologic_n$pathologic_n <- gsub("N2", "N+", pathologic_n$pathologic_n)

pathologic_n$pathologic_n <- gsub("N3", "N+", pathologic_n$pathologic_n)

N_0 <- pathologic_n[pathologic_n$pathologic_n == "N-",]

N_1 <- pathologic_n[pathologic_n$pathologic_n == "N+",]

pathologic_n <- rbind(N_0,N_1)

colnames(pathologic_n) <- c("C16orf74","clinical")

pathologic_n$clinical <- factor(pathologic_n$clinical, levels = c("N-","N+"))

comparisons <- list(c("N-","N+"))

e <- ggplot(pathologic_n, aes(x = clinical, y = C16orf74))

e + geom_boxplot()

e + geom_violin(trim = FALSE) +

stat_summary(

fun.data = "mean_sdl", fun.args = list(mult = 1),

geom = "pointrange", color = "black"

)

e + geom_violin(aes(fill = clinical), trim = FALSE) +

geom_boxplot(width = 0.5, fill = c("#EED67B", "#EC8874"), color = "black", alpha = 1)+

geom_jitter(shape = 16, position = position_jitter(0.2), color = "black", size = 1) +

scale_fill_manual(values = c("#F7EEC8", "#F7CCBE"))+

theme(legend.position = "none")+

stat_compare_means(comparisons = comparisons,method = "wilcox.test")

clinical_n <- rt[,c(1,15)]

clinical_n$clinical_n <- gsub("N0", "N-", clinical_n$clinical_n)

clinical_n$clinical_n <- gsub("N1", "N+", clinical_n$clinical_n)

clinical_n$clinical_n <- gsub("N2", "N+", clinical_n$clinical_n)

clinical_n$clinical_n <- gsub("N3", "N+", clinical_n$clinical_n)

N_0 <- clinical_n[clinical_n$clinical_n == "N-",]

N_1 <- clinical_n[clinical_n$clinical_n == "N+",]

clinical_n <- rbind(N_0,N_1)

colnames(clinical_n) <- c("C16orf74","clinical")

clinical_n$clinical <- factor(clinical_n$clinical, levels = c("N-","N+"))

comparisons <- list(c("N-","N+"))

e <- ggplot(clinical_n, aes(x = clinical, y = C16orf74))

e + geom_boxplot()

e + geom_violin(trim = FALSE) +

stat_summary(

fun.data = "mean_sdl", fun.args = list(mult = 1),

geom = "pointrange", color = "black"

)

e + geom_violin(aes(fill = clinical), trim = FALSE) +

geom_boxplot(width = 0.5, fill = c("#EED67B", "#EC8874"), color = "black", alpha = 1)+

geom_jitter(shape = 16, position = position_jitter(0.2), color = "black", size = 1) +

scale_fill_manual(values = c("#F7EEC8", "#F7CCBE"))+

theme(legend.position = "none")+

stat_compare_means(comparisons = comparisons,method = "wilcox.test")

pathologic_m <- rt[,c(1,12)]

pathologic_m$pathologic_m <- gsub("M0", "M-", pathologic_m$pathologic_m)

pathologic_m$pathologic_m <- gsub("M1", "M+", pathologic_m$pathologic_m)

M_0 <- pathologic_m[pathologic_m$pathologic_m == "M-",]

M_1 <- pathologic_m[pathologic_m$pathologic_m == "M+",]

pathologic_m <- rbind(M_0,M_1)

colnames(pathologic_m) <- c("C16orf74","clinical")

pathologic_m$clinical <- factor(pathologic_m$clinical, levels = c("M-","M+"))

comparisons <- list(c("M-","M+"))

e <- ggplot(pathologic_m, aes(x = clinical, y = C16orf74))

e + geom_boxplot()

e + geom_violin(trim = FALSE) +

stat_summary(

fun.data = "mean_sdl", fun.args = list(mult = 1),

geom = "pointrange", color = "black"

)

e + geom_violin(aes(fill = clinical), trim = FALSE) +

geom_boxplot(width = 0.5, fill = c("#EED67B", "#EC8874"), color = "black", alpha = 1)+

geom_jitter(shape = 16, position = position_jitter(0.2), color = "black", size = 1) +

scale_fill_manual(values = c("#F7EEC8", "#F7CCBE"))+

theme(legend.position = "none")+

stat_compare_means(comparisons = comparisons,method = "wilcox.test")

clinical_m <- rt[,c(1,16)]

clinical_m$clinical_m <- gsub("M0", "M-", clinical_m$clinical_m)

clinical_m$clinical_m <- gsub("M1", "M+", clinical_m$clinical_m)

M_0 <- clinical_m[clinical_m$clinical_m == "M-",]

M_1 <- clinical_m[clinical_m$clinical_m == "M+",]

clinical_m <- rbind(M_0,M_1)

colnames(clinical_m) <- c("C16orf74","clinical")

clinical_m$clinical <- factor(clinical_m$clinical, levels = c("M-","M+"))

comparisons <- list(c("M-","M+"))

e <- ggplot(clinical_m, aes(x = clinical, y = C16orf74))

e + geom_boxplot()

e + geom_violin(trim = FALSE) +

stat_summary(

fun.data = "mean_sdl", fun.args = list(mult = 1),

geom = "pointrange", color = "black"

)

e + geom_violin(aes(fill = clinical), trim = FALSE) +

geom_boxplot(width = 0.5, fill = c("#EED67B", "#EC8874"), color = "black", alpha = 1)+

geom_jitter(shape = 16, position = position_jitter(0.2), color = "black", size = 1) +

scale_fill_manual(values = c("#F7EEC8", "#F7CCBE"))+

theme(legend.position = "none")+

stat_compare_means(comparisons = comparisons,method = "wilcox.test")

rm(list = ls())

# if (!requireNamespace("BiocManager", quietly = TRUE))

# install.packages("BiocManager")

# BiocManager::install(version='devel')

#

# BiocManager::install("TCGAbiolinks")

# library(TCGAbiolinks)

# query <- GDCquery(

# project = "TCGA-HNSC",

# data.category = "Simple Nucleotide Variation",

# data.type = "Masked Somatic Mutation",

# access = "open"

# )

# GDCdownload(query)

# GDCprepare(query, save = T,save.filename = "output/TCGA-HNSC_SNP.Rdata")

rm(list = ls())

#BiocManager::install("maftools")

library(maftools)

library(export)

load(file = "output/TCGA-HNSC_SNP.Rdata")

new_data <- data %>%

group_by(Tumor_Sample_Barcode) %>%

summarise(Count = n()) %>%

mutate(ID=substr(Tumor_Sample_Barcode,1,12)) %>%

arrange(desc(Count)) %>%

distinct(ID,.keep_all = T)

index_Tumor_Sample_Barcode <- new_data$Tumor_Sample_Barcode

data <- subset(data,Tumor_Sample_Barcode %in% index_Tumor_Sample_Barcode)

data$ID <- substr(data$Tumor_Sample_Barcode,1,12)

load(file = "output/exprSet_vst.Rdata")

rt <- exprSet_vst %>%

column_to_rownames("ID") %>%

t() %>%

as.data.frame() %>%

rownames_to_column("TCGA_ID") %>%

dplyr::select(TCGA_ID,C16orf74) %>%

column_to_rownames("TCGA_ID")

index_all <- rownames(rt)

dat <- subset(data,ID %in% index_all)

index_res <- substr(dat$ID,1,12)

index_res <- unique(unlist(index_res))

rt <- rt %>%

mutate(TCGA_ID=rownames(rt)) %>%

dplyr::select(TCGA_ID,C16orf74)

rt <- rt[index_res,]

rownames(rt) <- NULL

rt <- rt %>%

column_to_rownames("TCGA_ID")

median_value <- median(rt$C16orf74, na.rm = TRUE)

rt$risk <- ifelse(rt$C16orf74 <= median_value, "low", "high")

high <- rt[rt$risk == "high",]

low <- rt[rt$risk == "low",]

index_high <- rownames(high)

dat <- subset(data,ID %in% index_high)

dat <- dplyr::select(dat,-ID)

maf.coad <- dat

maf <- read.maf(maf.coad)

oncoplot(maf = maf, top = 15,fontSize = 0.8)

tmb_high = tmb(maf = maf)

index_low <- rownames(low)

dat <- subset(data,ID %in% index_low)

dat <- dplyr::select(dat,-ID)

maf.coad <- dat

maf <- read.maf(maf.coad)

oncoplot(maf = maf, top = 15,fontSize = 0.8)

tmb_low = tmb(maf = maf)

tmb_high$Source <- 'high'

tmb_low$Source <- 'low'

combined_df <- rbind(tmb_low, tmb_high)

test_result <- wilcox.test(total_perMB_log ~ Source, data = combined_df, exact = FALSE)

print(test_result)

library(ggpubr)

library(ggplot2)

ggboxplot(

combined_df, x = "Source", y = "total_perMB_log",

color = "Source", palette = c("#2878b5", "#c82423"),

add = "jitter"

)+

stat_compare_means(method = "wilcox.test")

boxplot=ggviolin(combined_df, x="Source", y="total_perMB_log", fill="Source",

xlab="",

ylab="Tumor tmbation burden (log10)",

legend.title="",

palette = c("#2878b5","#c82423"),

add = "boxplot", add.params = list(fill="white"))+

stat_compare_means(method = "wilcox.test",label.x = 1.35,label.y = 3)

print(boxplot)

library(export)

load(file = "output/exprSet_vst.Rdata")

exprSet_vst <- exprSet_vst %>%

column_to_rownames("ID") %>%

t() %>%

as.data.frame() %>%

rownames_to_column("TCGA_ID") %>%

dplyr::select(TCGA_ID,C16orf74)

cli=read.table("resource/HNSC_clinical.txt",header=T,sep="\t",check.names=F,row.names=1)

sur=read.table("resource/HNSC_survival.txt",header=T,sep="\t",check.names=F,row.names=1)

colnames(sur)[2] <- "fustat"

colnames(sur)[1] <- "futime"

sur$futime <- sur$futime / 365

clinical_new <- cli %>%

rownames_to_column("TCGA_ID")

survival_new <- sur %>%

rownames_to_column("TCGA_ID")

clinical_final <- merge(clinical_new,survival_new,by="TCGA_ID")

clinical_final <- merge(clinical_final,exprSet_vst,by="TCGA_ID")

colnames(clinical_final)[1] <- "ID"

risk <- clinical_final %>%

dplyr::select(ID,C16orf74,futime,fustat)

combined_df$ID <- substr(combined_df$Tumor_Sample_Barcode,1,12)

combined_df <- combined_df %>%

arrange(desc(total_perMB)) %>%

distinct(ID,.keep_all = T) %>%

dplyr::select(ID,total_perMB)

data <- merge(risk,combined_df,by="ID")

colnames(data)[5] <- "TMB"

data <- data %>%

mutate(risk = if_else(C16orf74 <= median(C16orf74, na.rm = TRUE), "low", "high")) %>%

column_to_rownames("ID")

res.cut=surv_cutpoint(data, time = "futime", event = "fustat", variables =c("TMB"))

cutoff=as.numeric(res.cut$cutpoint[1])

tmbType=ifelse(data[,"TMB"]<=cutoff, "L-TMB", "H-TMB")

scoreType=ifelse(data$risk=="low", "L-Risk", "H-Risk")

mergeType=paste0(tmbType, " + ", scoreType)

bioSurvival=function(surData=null, outFile=null){

diff=survdiff(Surv(futime, fustat) ~ group, data=surData)

length=length(levels(factor(surData[,"group"])))

pValue=1-pchisq(diff$chisq, df=length-1)

if(pValue<0.001){

pValue="p<0.001"

}else{

pValue=paste0("p=",sprintf("%.03f",pValue))

}

fit <- survfit(Surv(futime, fustat) ~ group, data = surData)

bioCol=c("#FF0000","#0066FF","#6E568C","#7CC767","#223D6C","#D20A13","#FFD121","#088247","#11AA4D")

bioCol=bioCol[1:length]

surPlot=ggsurvplot(fit,

data=surData,

conf.int=F,

pval=pValue,

pval.size=6,

legend.title="Group",

legend.labs=levels(factor(surData[,"group"])),

font.legend=10,

legend = "top",

xlab="Time(years)",

break.time.by = 2,

palette = bioCol,

#surv.median.line = "hv",

risk.table=F,

cumevents=F,

risk.table.height=.25)

print(surPlot)

}

data$group <- tmbType

bioSurvival(surData = data)

data$group <- mergeType

bioSurvival(surData = data)

rm(list = ls())

#BiocManager::install("maftools")

library(maftools)

library(export)

load(file = "output/TCGA-HNSC_SNP.Rdata")

new_data <- data %>%

group_by(Tumor_Sample_Barcode) %>%

summarise(Count = n()) %>%

mutate(ID=substr(Tumor_Sample_Barcode,1,12)) %>%

arrange(desc(Count)) %>%

distinct(ID,.keep_all = T)

index_Tumor_Sample_Barcode <- new_data$Tumor_Sample_Barcode

data <- subset(data,Tumor_Sample_Barcode %in% index_Tumor_Sample_Barcode)

data$ID <- substr(data$Tumor_Sample_Barcode,1,12)

load(file = "output/exprSet_vst.Rdata")

rt <- exprSet_vst %>%

column_to_rownames("ID") %>%

t() %>%

as.data.frame() %>%

rownames_to_column("TCGA_ID") %>%

dplyr::select(TCGA_ID,C16orf74) %>%

column_to_rownames("TCGA_ID")

index_all <- rownames(rt)

dat <- subset(data,ID %in% index_all)

index_res <- substr(dat$ID,1,12)

index_res <- unique(unlist(index_res))

rt <- rt %>%

mutate(TCGA_ID=rownames(rt)) %>%

dplyr::select(TCGA_ID,C16orf74)

rt <- rt[index_res,]

rownames(rt) <- NULL

rt <- rt %>%

column_to_rownames("TCGA_ID")

median_value <- median(rt$C16orf74, na.rm = TRUE)

rt$risk <- ifelse(rt$C16orf74 <= median_value, "low", "high")

rt <- rt %>%

rownames_to_column("id")

high <- rt[rt$risk == "high",]

low <- rt[rt$risk == "low",]

index_high <- high[,"id"]

dat <- subset(data,ID %in% index_high)

dat <- dplyr::select(dat,-ID)

maf.coad <- dat

high_maf <- read.maf(maf.coad)

index_low <- low[,"id"]

dat <- subset(data,ID %in% index_low)

dat <- dplyr::select(dat,-ID)

maf.coad <- dat

low_maf <- read.maf(maf.coad)

pt.vs.rt <- mafCompare(m1 = high_maf, m2 = low_maf, m1Name = 'High', m2Name = 'Low', minMut = 10)

print(pt.vs.rt)

forestPlot(mafCompareRes = pt.vs.rt, pVal = 0.05, color = c('royalblue', 'maroon'), geneFontSize = 0.8)

library(export)

rm(list = ls())

load(file = "output/exprSet_vst.Rdata")

exprSet_vst <- exprSet_vst %>%

column_to_rownames("ID") %>%

t() %>%

as.data.frame() %>%

rownames_to_column("TCGA_ID") %>%

dplyr::select(TCGA_ID,C16orf74)

cli=read.table("resource/HNSC_clinical.txt",header=T,sep="\t",check.names=F,row.names=1)

sur=read.table("resource/HNSC_survival.txt",header=T,sep="\t",check.names=F,row.names=1)

colnames(sur)[2] <- "fustat"

colnames(sur)[1] <- "futime"

clinical_new <- cli %>%

rownames_to_column("TCGA_ID")

survival_new <- sur %>%

rownames_to_column("TCGA_ID")

clinical_final <- merge(clinical_new,survival_new,by="TCGA_ID")

clinical_final <- merge(clinical_final,exprSet_vst,by="TCGA_ID")

clinical_final$futime <- clinical_final$futime / 365

colnames(clinical_final)[19] <- "riskScore"

clinical <- clinical_final %>%

dplyr::select(TCGA_ID,futime,fustat,gender,age,

clinical_t,clinical_n,clinical_m,

clinical_stage,lymphovascular_invasion,perineural_invasion,riskScore) %>%

column_to_rownames("TCGA_ID")

clinical <- clinical[!(apply(clinical, 1, function(x) any(x == ""))), ]

clinical$clinical_t <- factor(clinical$clinical_t,levels = c("T1","T2","T3","T4"),order=T)

clinical$clinical_n <- factor(clinical$clinical_n,levels = c("N0","N1","N2","N3"),order=T)

clinical$clinical_m <- factor(clinical$clinical_m,levels = c("M0","M1"),order=T)

clinical$clinical_stage <- factor(clinical$clinical_stage,levels = c("I","II","III","IV"),order=T)

clinical$lymphovascular_invasion <- factor(clinical$lymphovascular_invasion,levels = c("NO","YES"),order=T)

clinical$perineural_invasion <- factor(clinical$perineural_invasion,levels = c("NO","YES"),order=T)

map_clinical_t <- setNames(1:4, c("T1", "T2", "T3", "T4"))

map_clinical_n <- setNames(1:4, c("N0", "N1", "N2","N3"))

map_clinical_m <- setNames(1:2, c("M0", "M1"))

map_clinical_stage <- setNames(4:1, c("I", "II", "III", "IV"))

map_invasion <- setNames(0:1, c("NO", "YES"))

clinical$clinical_t <- map_clinical_t[clinical$clinical_t]

clinical$clinical_n <- map_clinical_n[clinical$clinical_n]

clinical$clinical_m <- map_clinical_m[clinical$clinical_m]

clinical$clinical_stage <- map_clinical_stage[clinical$clinical_stage]

clinical$lymphovascular_invasion <- map_invasion[clinical$lymphovascular_invasion]

clinical$perineural_invasion <- map_invasion[clinical$perineural_invasion]

str(clinical)

clinical$fustat <- as.numeric(clinical$fustat)

clinical$age <- as.numeric(clinical$age)

clinical$clinical_stage <- as.numeric(clinical$clinical_stage)

clinical$lymphovascular_invasion <- as.numeric(clinical$lymphovascular_invasion)

clinical$perineural_invasion <- as.numeric(clinical$perineural_invasion)

clinical$clinical_t <- as.numeric(clinical$clinical_t)

clinical$clinical_n <- as.numeric(clinical$clinical_n)

clinical$clinical_m <- as.numeric(clinical$clinical_m)

str(clinical)

ddist <- datadist(clinical)

options(datadist="ddist")

units(clinical$futime) <- "year"

cox_cph <- cph(Surv(futime, fustat) ~ age+clinical_t+clinical_n+clinical_m+clinical_stage+

lymphovascular_invasion+perineural_invasion+riskScore,

x=T, y=T, surv=T,

data=clinical)

surv <- Survival(cox_cph)

cox_cph_nomogram<- nomogram(cox_cph,fun=list(function(x) surv(1, x), function(x) surv(3, x),function(x) surv(5, x)),##调整相应时间即可

lp= F,

funlabel=c('1-Year Survival','3-Year survival','5-Year Survival'),

maxscale=100,

fun.at=c('0.9','0.7','0.5','0.3','0.1'))

plot(cox_cph_nomogram,

lplabel="Linear Predictor",

xfrac = 0.2,

#varname.label = TRUE,

tcl = -0.2,

lmgp = 0.3,

points.label ='Points',

total.points.label = 'Total Points',

cap.labels = FALSE,

cex.var = 1,

cex.axis = 1,

col.grid = gray(c(0.8, 0.95)))

library(export)

Nomogram<-predict(cox_cph,clinical,type="lp")

clinical$Nomogram <- Nomogram

validate(cox_cph, method="boot", B=1000, dxy=T)

rcorrcens(Surv(futime,fustat) ~ predict(cox_cph), data = clinical)

library(rms)

dd <- datadist(clinical)

options(datadist="dd")

####

time=1

f_1 <- cph(Surv(futime, fustat) ~ age+clinical_t+clinical_n+clinical_m+clinical_stage+

lymphovascular_invasion+perineural_invasion+riskScore,

x=T, y=T, surv=T, data=clinical, time.inc=time)

P_1 <- calibrate(f_1, cmethod="KM", method="boot", u=time, m=95, B=1000)

plot(P_1,

add = F,

subtitles = F,

cex.subtitles = 0.8,

lwd = 2,

lty = 1,

errbar.col = "red",

xlim = c(0,1),

ylim = c(0,1),

xlab="Nomogram-Predicted Probability of 1, 3, 5-Year OS",

ylab="Actual 1, 3, 5-Year OS(proportion)",

col="red",

sub=F)

abline(0, 1, col = "black")

####

time=3

f_3 <- cph(Surv(futime, fustat) ~ age+clinical_t+clinical_n+clinical_m+clinical_stage+

lymphovascular_invasion+perineural_invasion+riskScore,

x=T, y=T, surv=T, data=clinical, time.inc=time)

P_3 <- calibrate(f_3, cmethod="KM", method="boot", u=time, m=95, B=1000)

plot(P_3,

add = T,

subtitles = F,

cex.subtitles = 0.8,

lwd = 2,

lty = 1,

errbar.col = "orange",

xlim = c(0,1),

ylim = c(0,1),

xlab="Nomogram-Predicted Probability of 1, 3, 5-Year OS",

ylab="Actual 1, 3, 5-Year OS(proportion)",

col="orange",

sub=F)

abline(0, 1, col = "black")

####

time=5

f_5 <- cph(Surv(futime, fustat) ~ age+clinical_t+clinical_n+clinical_m+clinical_stage+

lymphovascular_invasion+perineural_invasion+riskScore,

x=T, y=T, surv=T, data=clinical, time.inc=time)

P_5 <- calibrate(f_5, cmethod="KM", method="boot", u=time, m=95, B=1000)

plot(P_5,

add = T,

subtitles = F,

cex.subtitles = 0.8,

lwd = 2,

lty = 1,

errbar.col = "blue",

xlim = c(0,1),

ylim = c(0,1),

xlab="Nomogram-Predicted Probability of 1, 3, 5-Year OS",

ylab="Actual 1, 3, 5-Year OS(proportion)",

col="blue",

sub=F)

abline(0, 1, col = "black")

####

####

legend("bottomright",legend = c("1 year","3 year","5 year"),

col = c("red","orange","blue"),lwd = 2)

abline(0, 1, col = "black")

index_data <- clinical %>%

rownames_to_column("id") %>%

dplyr::select(id,futime,fustat,riskScore,Nomogram)

colnames(index_data)[2:3] <- c("time","status")

data <- index_data

library(riskRegression)

ddist <- datadist(data)

options(datadist = "ddist")

models = list(Riskscore=cph(Surv(time,status)~riskScore,data=data,x=TRUE,y=TRUE,surv = T),

Nomogram=cph(Surv(time,status)~Nomogram,data=data,x=TRUE,y=TRUE,surv = T)

)

times <- c(1,2,3,4,5,6,7,8,9,10)

cindex<- cindex(models,

formula=Surv(time,status)~1,

eval.times = times,

data=data)

plot(cindex)

cindex$AppCindex

cindex_df <- data.frame(

Time = times,

do.call(cbind,cindex$AppCindex)

)

cindex_df

dat = pivot_longer(cindex_df,cols = 2:ncol(cindex_df),

names_to = "model",

values_to = "cindex")

head(dat)

dat$model <- factor(dat$model, levels = c("Nomogram",

"Riskscore"

))

library(ggplot2)

ggplot(dat, aes(x = Time, y = cindex)) +

geom_line(aes(color = model),linewidth = 1.5) +

scale_color_brewer(palette = "Set1")+

ylim(0,1)+

labs(title = "Time-dependent C-index", x = "Time (years)", y = "C-index") +

theme_bw() +

scale_x_continuous(breaks = seq(0, 20, by = 2))

library(export)

var="Nomogram"

ROC_rt=timeROC(T=clinical$futime, delta=clinical$fustat,

marker=clinical[,var], cause=1,

weighting='aalen',

times=c(1,3,5), ROC=TRUE)

plot(ROC_rt,time=1,col='green',title=FALSE,lwd=2)

plot(ROC_rt,time=3,col='blue',add=TRUE,title=FALSE,lwd=2)

plot(ROC_rt,time=5,col='red',add=TRUE,title=FALSE,lwd=2)

legend('bottomright',

c(paste0('AUC at 1 years: ',sprintf("%.03f",ROC_rt$AUC[1])),

paste0('AUC at 3 years: ',sprintf("%.03f",ROC_rt$AUC[2])),

paste0('AUC at 5 years: ',sprintf("%.03f",ROC_rt$AUC[3]))),

col=c("green",'blue','red'),lwd=2,bty = 'n')

median_score <- median(clinical$Nomogram)

clinical$Nomogram <- ifelse(clinical$Nomogram <= median_score, "low", "high")

genes <- "Nomogram"

your.surv <- Surv(clinical$futime, clinical$fustat)

your.km.plot <- function(genes,data){

print(genes)

group <- clinical[,genes]

survival_dat <- data.frame(group = group)

group <- factor(group, levels = c("low", "high"))

fit <- survfit(your.surv ~ group)

sdf <- survdiff(your.surv ~ group,rho=0)

p.val <- 1 - pchisq(sdf$chisq, length(sdf$n)-1)

p.val

photo2 <- ggsurvplot(fit,data = survival_dat,

legend.title = genes,

legend.labs = c("low","high"),

#legend = "top",

pval = T,

#pval.method = TRUE,

conf.int = TRUE,

risk.table = F,

#risk.table.col = "strata",

risk.table.y.text = F,

#linetype = "strata",

#surv.median.line = "hv",

xlab = "Time in years",

xlim = c(0,max(clinical$futime)+1),

break.time.by = 2,

size = 1.5,

#ggtheme = theme_bw(),

palette = c("#2878b5", "#c82423")

)

photo2

}

##2.

your.km.plot("Nomogram",data = clinical)

rm(list = ls())

load(file = "output/exprSet_vst.Rdata")

exprSet_vst <- exprSet_vst %>%

column_to_rownames("ID") %>%

t() %>%

as.data.frame() %>%

rownames_to_column("ID") %>%

dplyr::select(ID,C16orf74)

OS=read.table("resource/HNSC_survival.txt",header=T,sep="\t",check.names=F,row.names=1)

DFS=read.table("resource/HNSC_DFS.txt",header=T,sep="\t",check.names=F,row.names=1)

OS <- OS %>%

rownames_to_column("ID")

DFS <- DFS %>%

rownames_to_column("ID")

index_os <- OS$ID

index_dfs <- DFS$ID

DFS_0 <- OS %>% filter(!(ID %in% index_dfs))

colnames(DFS_0)[2] <- "dfs.time"

colnames(DFS_0)[3] <- "dfs"

DFS_final <- rbind(DFS_0,DFS)

colnames(DFS_final)[2] <- "DFS.time"

colnames(DFS_final)[3] <- "DFS"

OS_final <- merge(exprSet_vst,OS)

OS_final$OS.time <- OS_final$OS.time / 365

median_value <- median(OS_final$C16orf74, na.rm = TRUE)

OS_final$C16orf74 <- ifelse(OS_final$C16orf74 <= median_value, "low", "high")

OS_final <- OS_final %>%

column_to_rownames("ID") %>%

dplyr::select(OS.time,OS,C16orf74)

DFS_final <- merge(exprSet_vst,DFS_final)

DFS_final$DFS.time <- DFS_final$DFS.time / 365

median_value <- median(DFS_final$C16orf74, na.rm = TRUE)

DFS_final$C16orf74 <- ifelse(DFS_final$C16orf74 <= median_value, "low", "high")

DFS_final <- DFS_final %>%

column_to_rownames("ID") %>%

dplyr::select(DFS.time,DFS,C16orf74)

##1.写函数

genes <- "C16orf74"

your.surv <- Surv(OS_final$OS.time, OS_final$OS)

your.km.plot <- function(genes,data){

print(genes)

group <- OS_final[,genes] #分组

survival_dat <- data.frame(group = group)

group <- factor(group, levels = c("low", "high"))

fit <- survfit(your.surv ~ group)

sdf <- survdiff(your.surv ~ group,rho=0)

p.val <- 1 - pchisq(sdf$chisq, length(sdf$n)-1)

p.val

photo2 <- ggsurvplot(fit,data = survival_dat, #

legend.title = genes,#

legend.labs = c("low","high"), #

#legend = "top",#

pval = T, #

#pval.method = TRUE,#

conf.int = TRUE,#

risk.table = F, #

#risk.table.col = "strata", #

risk.table.y.text = F,#

#linetype = "strata", #

#surv.median.line = "hv", #

xlab = "Time in years", #

xlim = c(0,max(OS_final$OS.time)+1), #

break.time.by = 2, #

size = 1.5, #

#ggtheme = theme_bw(), #

palette = c("#2878b5", "#c82423")#

)

photo2 #

}

your.km.plot("C16orf74",data = OS_final)

genes <- "C16orf74"

your.surv <- Surv(DFS_final$DFS.time, DFS_final$DFS)

your.km.plot <- function(genes,data){

print(genes)

group <- DFS_final[,genes] #分组

survival_dat <- data.frame(group = group)

group <- factor(group, levels = c("low", "high"))

fit <- survfit(your.surv ~ group)

sdf <- survdiff(your.surv ~ group,rho=0)

p.val <- 1 - pchisq(sdf$chisq, length(sdf$n)-1)

p.val

photo2 <- ggsurvplot(fit,data = survival_dat, #

legend.title = genes,#

legend.labs = c("low","high"), #

#legend = "top",#

pval = T, #

#pval.method = TRUE,#

conf.int = TRUE,#

risk.table = F, #

#risk.table.col = "strata", #

risk.table.y.text = F,#

#linetype = "strata", #

#surv.median.line = "hv", #

xlab = "Time in years", #

xlim = c(0,max(DFS_final$DFS.time)+1), #

break.time.by = 2, #

size = 1.5, #

#ggtheme = theme_bw(), #

palette = c("#2878b5", "#c82423")#

)

photo2

}

##2.

your.km.plot("C16orf74",data = DFS_final)

rm(list = ls())

library(tidyr)

library(dplyr)

library(tibble)

library(survminer)

library(survival)

library(ggplot2)

library(ggprism)

library(tidyverse)

library(timeROC)

library(data.table)

library(pheatmap)

library(ggpubr)

library(rms)

library(foreign)

library(utils)

library(limma)

library(estimate)

library(WGCNA)

library(DESeq2)

library(GSVA)

library(matrixStats)

library(cowplot)

library(ggExtra)

# install.packages("estimate", repos="http://R-Forge.R-project.org")

# install.packages("estimate_1.0.13.tar.gz",repos = NULL,type = "source")

# options("repos"=c(CRAN="https://mirrors.tuna.tsinghua.edu.cn/CRAN/"))

# options(BioC_mirror="https://mirrors.tuna.tsinghua.edu.cn/bioconductor")

# if(!require("data.table")) install.packages("data.table",update = F,ask = F)

# if(!require("GSVA")) BiocManager::install("GSVA",update = F,ask = F)

# if(!require("matrixStats")) BiocManager::install("matrixStats",update = F,ask = F)

rm(list = ls())

load(file = "resource/gene_id_tcga_v36.Rdata")

HNSC <- readRDS("resource/TCGA.rds/TCGA-HNSC.rds")

HNSC <- as.data.frame(HNSC)

HNSC <- HNSC %>%

rownames_to_column("ID")

HNSC <- HNSC %>%

column_to_rownames("ID") %>%

t() %>%

as.data.frame() %>%

mutate(newcolumn = rowMeans(.)) %>%

arrange(desc(newcolumn))

HNSC$sample <- substr(rownames(HNSC),14,15)

HNSC$ID <- substr(rownames(HNSC),1,12)

HNSC <- HNSC %>%

rownames_to_column("TCGA_ID")

HNSC <- HNSC %>%

filter(sample=="01") %>%

dplyr::select(-sample) %>%

dplyr::select(-TCGA_ID) %>%

dplyr::select(-newcolumn) %>%

dplyr::select(ID,everything()) %>%

distinct(ID,.keep_all = T)

HNSC <- HNSC %>%

column_to_rownames("ID") %>%

t() %>%

as.data.frame() %>%

rownames_to_column("gene_id")

expression <- merge(gene_id,HNSC,by="gene_id")

expression <- expression %>%

filter(gene_type=="protein_coding") %>%

dplyr::select(-gene_type) %>%

dplyr::select(-gene_id) %>%

mutate(newcolumn = rowMeans(.[,-1])) %>%

arrange(desc(newcolumn)) %>%

distinct(gene_name,.keep_all = T) %>%

dplyr::select(-newcolumn) %>%

column_to_rownames("gene_name")

exptDesign_TCGA <- data.frame(

condition = rep("tumor", length(colnames(expression))),

row.names = colnames(expression)

)

dds <- DESeqDataSetFromMatrix(

countData = expression,

colData = exptDesign_TCGA,

design = ~ 1)

nrow(dds)

rownames(dds)

dds <- dds[rowMedians(counts(dds))>0,]

nrow(dds)

system.time(vsd <- vst(dds, blind = FALSE))

exprSet_vst <- as.data.frame(assay(vsd))

exprSet_vst <- exprSet_vst %>%

t() %>%

as.data.frame()

exprSet_vst <- exprSet_vst %>%

rownames_to_column("ID")

risk <- exprSet_vst %>%

dplyr::select(ID,C16orf74)

colnames(risk)[2] <- "riskScore"

exprSet_vst <- merge(exprSet_vst,risk,by="ID")

exprSet_vst <- exprSet_vst %>%

column_to_rownames("ID") %>%

dplyr::select(-riskScore)

sample <- exprSet_vst %>%

rownames_to_column("ID") %>%

dplyr::select(ID)

exprSet_vst <- exprSet_vst %>%

t() %>%

as.data.frame()

risk <- merge(sample,risk,by="ID")

median_value <- median(risk$riskScore, na.rm = TRUE)

risk$riskScore <- ifelse(risk$riskScore <= median_value, "low", "high")

metadata <- risk

colnames(metadata)[2] <- "group"

colnames(metadata)[1] <- "TCGA_ID"

data <- exprSet_vst %>%

rownames_to_column("ID")

rm(HNSC)

rm(dds)

rm(expression)

rm(exptDesign_TCGA)

rm(gene_id)

rm(risk)

rm(metadata)

rm(vsd)

rm(median_value)

rm(sample)

write.table(data, file="output/uniq.symbol.txt", sep="\t", quote=F, col.names=T, row.names = F)

####

filterCommonGenes(input.f="output/uniq.symbol.txt",

output.f="output/commonGenes.gct",

id="GeneSymbol")

####

estimateScore(input.ds="output/commonGenes.gct",

output.ds="output/estimateScore.gct")

####

scores <- read.table("output/estimateScore.gct", skip=2, header=T, check.names=F)

rownames(scores) <- scores[,1]

scores <- t(scores[,3:ncol(scores)])

rownames(scores) <- gsub("\\.", "\\-", rownames(scores))

scores <- as.data.frame(scores[,1:3])

scores <- scores %>%

rownames_to_column("ID")

data_C16orf74 <- data %>%

column_to_rownames("ID") %>%

t() %>%

as.data.frame() %>%

rownames_to_column("ID") %>%

dplyr::select(ID,C16orf74)

data <- merge(data_C16orf74,scores,by="ID")

colnames(data)[2] <- "riskScore"

median_riskScore <- median(data$riskScore, na.rm = TRUE)

data$risk <- ifelse(data$riskScore >= median_riskScore, 'high', 'low')

data <- data %>%

column_to_rownames("ID") %>%

dplyr::select(riskScore,risk,everything())

####

data <- data[,-1]

data <- data %>%

pivot_longer(cols=-1,

names_to= "gene",

values_to = "Score")

ggplot(data = data,aes(x=gene,y=Score,fill=risk))+

geom_boxplot()+

theme_bw()+

stat_compare_means(label = "p.format")+

scale_fill_manual(values = c("low" = "#00BFC4", "high" = "#F8766D"))

library(export)

graph2ppt(file = "output/Cibersort",width=5,height=4.5)

load(file = "resource/cellMarker_ssGSEA.Rdata")

##加载表达矩阵

data <- exprSet_vst

data <- as.matrix(data)

gsva_data <- gsva(gsvaParam(as.matrix(data), cellMarker))

#Error: [matrixStats (>= 1.2.0)] useNames = NA is defunct.

#Instead, specify either useNames = TRUE or useNames = FALSE. See also ?

#matrixStats::matrixStats.options

#remotes::install_version("matrixStats", version="1.1.0")

#restart your session and run previous scripts

pheatmap(gsva_data, #

cluster_rows = F,#

cluster_cols = F,#

##annotation_col =annotation_col, #

annotation_legend=TRUE, #

show_rownames = T,#

show_colnames = F,#

##scale = "row", #

color =colorRampPalette(c("blue", "white","red"))(100),#

#filename = "heatmap_F.pdf",#

cellwidth = 1, cellheight = 10,#

fontsize = 10)

risk <- data_C16orf74

colnames(risk)[2] <- "riskScore"

heatdata <- gsva_data %>%

t() %>%

as.data.frame() %>%

rownames_to_column("ID")

heatdata <- merge(risk,heatdata,by="ID")

rt <- heatdata %>%

column_to_rownames("ID") %>%

dplyr::select(-riskScore) %>%

t() %>%

as.data.frame()

Type <- heatdata %>%

column_to_rownames("ID") %>%

dplyr::select(riskScore)

median_riskScore <- median(Type$riskScore, na.rm = TRUE)

Type$risk <- ifelse(Type$riskScore >= median_riskScore, 'high', 'low')

Type$risk <- factor(Type$risk, levels = c('low', 'high'))

Type <- Type %>%

dplyr::select(risk,riskScore)

var="riskScore"

Type=Type[order(Type[,var]),]

rt=rt[,row.names(Type)]

ann_colors <- list(risk = c(low = "#2878b5", high = "#c82423"),

riskScore = colorRampPalette(c("#2878b5", "white", "#c82423"))(100)

)

pheatmap(rt, annotation=Type,

color =colorRampPalette(c("blue", "white","red"))(100),

cluster_rows = FALSE,

cluster_cols = FALSE,

scale="row",

show_colnames=FALSE,

fontsize=8,

fontsize_row=7,

fontsize_col=3,

cellwidth = 0.8,

cellheight = 15,

annotation_colors = ann_colors)

library(export)

save(heatdata,file = "output/ssGSVA.Rdata")

median_riskScore <- median(heatdata$riskScore, na.rm = TRUE)

heatdata$risk <- ifelse(heatdata$riskScore >= median_riskScore, 'high', 'low')

heatdata$risk <- factor(heatdata$risk, levels = c('low', 'high'))

heatdata <- heatdata %>%

dplyr::select(riskScore,risk,everything()) %>%

column_to_rownames("ID")

####

dd1 <- heatdata %>%

pivot_longer(cols=3:30,

names_to= "celltype",

values_to = "Score")

dd1$celltype <- factor(dd1$celltype,levels = c("Eosinophil","Immature B cell","Mast cell","T follicular helper cell","Activated B cell",

"Plasmacytoid dendritic cell","Immature dendritic cell","Central memory CD4 T cell",

"MDSC","Activated CD8 T cell","Monocyte","Effector memeory CD8 T cell","Macrophage",

"Activated dendritic cell","Effector memeory CD4 T cell","Natural killer cell",

"Regulatory T cell","Type 1 T helper cell","Central memory CD8 T cell",

"Type 17 T helper cell","Gamma delta T cell","Natural killer T cell",

"CD56bright natural killer cell","CD56dim natural killer cell","Neutrophil",

"Activated CD4 T cell","Type 2 T helper cell","Memory B cell"),ordered = F)

library(ggplot2)

library(ggpubr)

ggplot(data =dd1, aes(x = celltype, y = Score))+

geom_boxplot(aes(fill = risk),position = position_dodge(1),width=.3,outlier.shape = NA)+

geom_violin(aes(colour = risk),position = position_dodge(1),scale = "width",fill=NA)+

theme_bw()+

theme(axis.text.x = element_text(angle = 45, hjust = 1,vjust = 1, colour = "black"))+

stat_compare_means(aes(group=risk), label = "p.signif")+

scale_fill_manual(values = c("low" = "#00BFC4", "high" = "#F8766D"))

library(export)

graph2ppt(file = "output/ssGSEA",width=19,height=7)

ICB <- data.table::fread("resource/ICB.txt",data.table = F)

ICB_data <- exprSet_vst %>%

rownames_to_column("gene")

ICB_data <- merge(ICB,ICB_data,by="gene")

ICB_data <- ICB_data %>%

column_to_rownames("gene") %>%

t() %>%

as.data.frame()

ICB_risk <- heatdata %>%

dplyr::select(riskScore,risk)

ICB_final <- merge(ICB_risk,ICB_data,by = "row.names", all = F)

ICB_final <- ICB_final %>%

column_to_rownames("Row.names")

####

dd2 <- ICB_final %>%

pivot_longer(cols=3:ncol(ICB_final),

names_to= "ICB",

values_to = "expression")

ICB_name <- colnames(ICB_data)

dd2$ICB <- factor(dd2$ICB,levels = ICB_name,ordered = F)

library(ggplot2)

library(ggpubr)

ggplot(data =dd2, aes(x = ICB, y = expression))+

geom_boxplot(aes(fill = risk),position = position_dodge(1),width=.3,outlier.shape = NA)+

geom_violin(aes(colour = risk),position = position_dodge(1),scale = "width",fill=NA)+

theme_bw()+

theme(axis.text.x = element_text(angle = 45, hjust = 1,vjust = 1, colour = "black"))+

stat_compare_means(aes(group=risk), label = "p.signif")+

scale_fill_manual(values = c("low" = "#00BFC4", "high" = "#F8766D"))

library(export)

dd1 <- ICB$gene

dd1

ICB_index <- c("ADORA2A","BTLA","BTN2A1","BTN2A2","BTN3A1","BTNL9","CD160","CD209","CD226",

"CD27","CD274","CD276","CD28","CD40","CD40LG","CD47","CD70","CD80","CD86",

"CD96","CEACAM1","CTLA4","HAVCR2","HLA-DMA","HLA-DMB","HLA-DOA","HLA-DOB",

"HLA-DPA1","HLA-DPB1","HLA-DQA1","HLA-DQB1","HLA-DRA","HLA-DRB1",

"HLA-DRB5","HLA-G","ICOS","ICOSLG","IDO1","KIR2DL1","KIR2DL3","KIR2DL4",

"KIR3DL1","KIR3DL2","LAG3","LGALS9",

"PDCD1","PDCD1LG2","PVR","TIGIT","TNFRSF14",

"TNFRSF4","TNFRSF9","TNFSF14","TNFSF4","TNFSF9")

ICB_data <- ICB_data[,ICB_index]

ICB_final <- ICB_final %>%

dplyr::select(1:2)

ICB_final <- merge(ICB_risk,ICB_data,by = "row.names", all = F)

ICB_final <- ICB_final %>%

column_to_rownames("Row.names")

dd2 <- ICB_final %>%

pivot_longer(cols=3:ncol(ICB_final),

names_to= "ICB",

values_to = "expression")

ICB_name <- colnames(ICB_data)

dd2$ICB <- factor(dd2$ICB,levels = ICB_name,ordered = F)

library(ggplot2)

library(ggpubr)

ggplot(data =dd2, aes(x = ICB, y = expression))+

geom_boxplot(aes(fill = risk),position = position_dodge(1),width=.3,outlier.shape = NA)+

geom_violin(aes(colour = risk),position = position_dodge(1),scale = "width",fill=NA)+

theme_bw()+

theme(axis.text.x = element_text(angle = 45, hjust = 1,vjust = 1, colour = "black"))+

stat_compare_means(aes(group=risk), label = "p.signif")+

scale_fill_manual(values = c("low" = "#00BFC4", "high" = "#F8766D"))

exprSet1 <- as.data.frame(t(heatdata))

gene <- "riskScore"

####

batch_cor <- function(gene){

y <- as.numeric(exprSet1[gene,])

rownames <- rownames(exprSet1)[3:30]

do.call(rbind,future_lapply(rownames, function(x){

dd <- cor.test(as.numeric(exprSet1[x,]),y,method="spearman")

data.frame(gene=gene,mRNAs=x,cor=dd$estimate,p.value=dd$p.value )

}))

}

library(future.apply)

# plan(multiprocess)

system.time(dd <- batch_cor(gene))

####

library(ggpubr)

library(reshape)

write.table(dd,file="output/dd.xls",sep="\t",row.names=F,quote=F)

bc <- read.table("output/dd.xls",header = T,sep="\t",check.names=F)

bc <- bc %>%

arrange(cor)

cor_index <- bc$mRNAs

bc$Cor <- abs(bc$cor)

library(tibble)

library(dplyr)

# bc$mRNAs <- factor(bc$mRNAs,levels = c("Effector memeory CD4 T cell",

# "Type 2 T helper cell",

# "Activated CD4 T cell",

# "Immature dendritic cell",

# "Effector memeory CD8 T cell",

# "Immature B cell",

# "Effector memeory CD8 T cell",

# "Activated CD8 T cell",

# "Type 1 T helper cell",

# "Macrophage",

# "Activated B cell",

# "Gamma delta T cell",

# "Natural killer T cell",

# "Activated CD4 T cell",

# "CD56bright natural killer cell",

# "Natural killer cell",

# "Central memory CD8 T cell",

# "Effector memeory CD4 T cell",

# "Immature dendritic cell",

# "Mast cell",

# "Plasmacytoid dendritic cell",

# "Type 17 T helper cell",

# "Monocyte",

# "Neutrophil",

# "Eosinophil",

# "CD56dim natural killer cell",

# "Type 2 T helper cell",

# "Memory B cell"),ordered = F)

bc$mRNAs <- factor(bc$mRNAs,levels = cor_index,ordered = F)

ggplot(bc, aes(mRNAs, cor),rotate = T) +

geom_segment(aes(xend=mRNAs, yend = 0),linetype = "dashed",colour = "orange") +

geom_point(aes(color = p.value, size = Cor)) +

scale_color_viridis_c(guide=guide_colorbar(reverse=TRUE)) +

scale_color_continuous(low="brown1", high="cyan", guide=guide_colorbar(reverse=TRUE))+

scale_size_continuous(range=c(2, 8)) +

theme_minimal() +

xlab("Cor") +

ylab(NULL)+

theme(axis.text.x = element_text(angle = 45, hjust = 1,vjust = 1, colour = "black"))

rm(list = ls())

load(file = "output/ssGSVA.Rdata")

data <- heatdata %>%

column_to_rownames("ID")

colnames(data) <- gsub(" ","_",colnames(data))

ggplot(data,aes(riskScore,Type_2_T_helper_cell))+

geom_point(col="#984ea3",size=3,alpha=0.7,stroke=1)+

geom_smooth(method=lm, se=T,na.rm=T, fullrange=T,size=1.5,col="#fdc086")+

geom_rug(col="#7fc97f")+

stat_cor(method = "pearson", digits = 3, size=5)+

theme_bw()+

theme(

plot.title = element_text(hjust = 0.5),

plot.margin = margin(1, 1, 1, 1, "cm"),

axis.title = element_text(size = 15),

axis.text = element_text(size = 15)

)

rm(list = ls())

load(file = "output/ssGSVA.Rdata")

sur=read.table("resource/HNSC_survival.txt",header=T,sep="\t",check.names=F,row.names=1)

colnames(heatdata) <- gsub(" ", "_", colnames(heatdata))

colnames(sur)[2] <- "fustat"

colnames(sur)[1] <- "futime"

sur$futime <- sur$futime / 365

library(tibble)

library(dplyr)

tcga_gsva2 <- sur %>%

rownames_to_column("ID") %>%

inner_join(.,heatdata, by = "ID") %>%

column_to_rownames("ID") %>%

select(-"riskScore") %>%

select("futime","fustat",everything())

library(survival)

library(survminer)

exprSet <- tcga_gsva2

# ####

genes <- "Natural_killer_T_cell"

data <- exprSet %>%

dplyr::select(futime,fustat,all_of(genes))

colnames(data)[1:2] <- c("OS.time","OS")

res.cut <- surv_cutpoint(data, time = "OS.time",

event = "OS",

variables = names(data)[3:ncol(data)],

minprop = 0.3) #

res.cat <- surv_categorize(res.cut)

summary(res.cut)

str(res.cat)

your.surv <- Surv(res.cat$OS.time, res.cat$OS)

your.km.plot <- function(genes,data){

print(genes)

group <- res.cat[,genes] #

survival_dat <- data.frame(group = group)

group <- factor(group, levels = c("low", "high"))

fit <- survfit(your.surv ~ group)

sdf <- survdiff(your.surv ~ group,rho=0)

p.val <- 1 - pchisq(sdf$chisq, length(sdf$n)-1)

p.val

photo2 <- ggsurvplot(fit,data = survival_dat, #

legend.title = genes,#

legend.labs = c("low","high"), #

#legend = "top",#

pval = T, #

#pval.method = TRUE,#

conf.int = TRUE,#

risk.table = F, #

#risk.table.col = "strata", #

risk.table.y.text = F,#

#linetype = "strata", #

#surv.median.line = "hv", #

xlab = "Time in years", #

xlim = c(0,max(res.cat$OS.time)+1), #

break.time.by = 2, #

size = 1.5, #

#ggtheme = theme_bw(), #

palette = c("#2878b5", "#c82423")#

)

photo2

}

##2.

your.km.plot(genes)

rm(list = ls())

library(tidyr)

library(dplyr)

library(tibble)

library(survminer)

library(survival)

library(ggplot2)

library(ggprism)

library(tidyverse)

library(timeROC)

library(data.table)

library(pheatmap)

library(ggpubr)

library(WGCNA)

library(DESeq2)

library(clusterProfiler)

library(org.Hs.eg.db)

rm(list = ls())

load(file = "resource/gene_id_tcga_v36.Rdata")

HNSC <- readRDS("resource/TCGA.rds/TCGA-HNSC.rds")

HNSC <- as.data.frame(HNSC)

HNSC <- HNSC %>%

rownames_to_column("ID")

HNSC <- HNSC %>%

column_to_rownames("ID") %>%

t() %>%

as.data.frame() %>%

mutate(newcolumn = rowMeans(.)) %>%

arrange(desc(newcolumn))

HNSC$sample <- substr(rownames(HNSC),14,15)

HNSC$ID <- substr(rownames(HNSC),1,12)

HNSC <- HNSC %>%

rownames_to_column("TCGA_ID")

HNSC <- HNSC %>%

filter(sample=="01") %>%

dplyr::select(-sample) %>%

dplyr::select(-TCGA_ID) %>%

dplyr::select(-newcolumn) %>%

dplyr::select(ID,everything()) %>%

distinct(ID,.keep_all = T)

HNSC <- HNSC %>%

column_to_rownames("ID") %>%

t() %>%

as.data.frame() %>%

rownames_to_column("gene_id")

expression <- merge(gene_id,HNSC,by="gene_id")

expression <- expression %>%

filter(gene_type=="protein_coding") %>%

dplyr::select(-gene_type) %>%

dplyr::select(-gene_id) %>%

mutate(newcolumn = rowMeans(.[,-1])) %>%

arrange(desc(newcolumn)) %>%

distinct(gene_name,.keep_all = T) %>%

dplyr::select(-newcolumn) %>%

column_to_rownames("gene_name")

exptDesign_TCGA <- data.frame(

condition = rep("tumor", length(colnames(expression))),

row.names = colnames(expression)

)

dds <- DESeqDataSetFromMatrix(

countData = expression,

colData = exptDesign_TCGA,

design = ~ 1)

nrow(dds)

rownames(dds)

dds <- dds[rowMedians(counts(dds))>0,]

nrow(dds)

system.time(vsd <- vst(dds, blind = FALSE))

exprSet_vst <- as.data.frame(assay(vsd))

exprSet_vst <- exprSet_vst %>%

t() %>%

as.data.frame()

exprSet_vst <- exprSet_vst %>%

rownames_to_column("ID")

risk <- exprSet_vst %>%

dplyr::select(ID,C16orf74)

colnames(risk)[2] <- "riskScore"

exprSet_vst <- exprSet_vst %>%

column_to_rownames("ID") %>%

t() %>%

as.data.frame()

datExpr0 <- exprSet_vst

gsg = goodSamplesGenes(datExpr0, verbose = 3)

gsg$allOK

if (!gsg$allOK){

# Optionally, print the gene and sample names that were removed:

if (sum(!gsg$goodGenes)>0)

#printFlush(paste("Removing genes:", paste(names(datExpr0)[!gsg$goodGenes], collapse = ", ")));

if (sum(!gsg$goodSamples)>0)

printFlush(paste("Removing samples:", paste(rownames(datExpr0)[!gsg$goodSamples], collapse = ", ")));

# Remove the offending genes and samples from the data:

datExpr0 = datExpr0[gsg$goodSamples, gsg$goodGenes]

}

datExpr <- datExpr0 %>%

t() %>%

as.data.frame()

median_value <- median(risk$riskScore, na.rm = TRUE)

risk$riskScore <- ifelse(risk$riskScore <= median_value, "low", "high")

colnames(risk)[1] <- "ID"

colnames(risk)[2] <- "condition"

enableWGCNAThreads()

powers = c(c(1:10), seq(from = 12, to=30, by=2))

length(powers)

sft = pickSoftThreshold(datExpr, powerVector = powers,networkType="signed", verbose = 5)

test <- sft$fitIndices

sizeGrWindow(9, 5)

par(mfrow = c(1,2))

cex1 = 0.9

# Scale-free topology fit index as a function of the soft-thresholding power

plot(sft$fitIndices[,1], -sign(sft$fitIndices[,3])*sft$fitIndices[,2],

xlab="Soft Threshold (power)",ylab="Scale Free Topology Model Fit,signed R^2",type="n",

main = paste("Scale independence"));

text(sft$fitIndices[,1], -sign(sft$fitIndices[,3])*sft$fitIndices[,2],

labels=powers,cex=cex1,col="red")

# this line corresponds to using an R^2 cut-off of h

abline(h=0.9,col="red")

# Mean connectivity as a function of the soft-thresholding power

plot(sft$fitIndices[,1], sft$fitIndices[,5],

xlab="Soft Threshold (power)",ylab="Mean Connectivity", type="n",

main = paste("Mean connectivity"))

text(sft$fitIndices[,1], sft$fitIndices[,5], labels=powers, cex=cex1,col="red")

cor <- WGCNA::cor

net = blockwiseModules(datExpr, power = 12, maxBlockSize = 20000,

TOMType = "unsigned", minModuleSize = 50,

networkType="signed",

reassignThreshold = 0, mergeCutHeight = 0.25,

numericLabels = TRUE, pamRespectsDendro = FALSE,

saveTOMs = TRUE,

saveTOMFileBase = "yourname",

verbose = 3)

cor<-stats::cor

table(net$colors)

sizeGrWindow(12, 9)

mergedColors = labels2colors(net$colors)

table(mergedColors)

plot(net$dendrograms[[1]])

plotDendroAndColors(net$dendrograms[[1]],

mergedColors[net$blockGenes[[1]]],

"Module colors",

dendroLabels = FALSE, hang = 0.03,

addGuide = TRUE, guideHang = 0.05)

moduleLabels = net$colors

moduleColors = labels2colors(net$colors)

geneTree = net$dendrograms[[1]]

test <- moduleEigengenes(datExpr, moduleColors)

MEs0 = moduleEigengenes(datExpr, moduleColors)$eigengenes

MEs = orderMEs(MEs0)

datTraits <- risk

datTraits$condition <- factor(datTraits$condition,levels = c("low","high"))

design=model.matrix(~0+ datTraits$condition)

design <- as.data.frame(design)

colnames(design)=levels(datTraits$condition)

moduleTraitCor = cor(MEs, design, use = "p")

nSamples <- nrow(datExpr)

moduleTraitPvalue = corPvalueStudent(moduleTraitCor, nSamples)

sizeGrWindow(10,6)

textMatrix = paste(signif(moduleTraitCor, 2), "(",

signif(moduleTraitPvalue, 1), ")", sep = "");

dim(textMatrix) = dim(moduleTraitCor)

par(mar = c(6, 8.5, 3, 3));

labeledHeatmap(Matrix = moduleTraitCor,

xLabels = colnames(design),

yLabels = names(MEs),

ySymbols = names(MEs),

colorLabels = FALSE,

colors = blueWhiteRed(50),

textMatrix = textMatrix,

setStdMargins = FALSE,

cex.text = 0.5,

zlim = c(-1,1),

main = paste("Module-trait relationships"))

dd1 <- data.frame(ME=names(MEs),color=substring(names(MEs),3))

dd2 <- data.frame(table(moduleColors))

colnames(dd2) <- c("color","num")

dd3 <- merge(dd1,dd2,by="color")

rownames(dd3) <- dd3$ME

dd3 <- dd3[names(MEs),]

par(mar = c(6, 5, 3, 3))

ynumbers <- paste0(names(MEs),paste0("(",dd3$num,")"))

ynumbers

labeledHeatmap(Matrix = moduleTraitCor,

xLabels = colnames(design),

yLabels = names(MEs),

ySymbols = ynumbers,

colorLabels = FALSE,

colors = blueWhiteRed(50),

textMatrix = textMatrix,

setStdMargins = FALSE,

cex.text = 0.8,

zlim = c(-1,1),

main = paste("Module-trait relationships"))

module = "yellow"

moduleGenes = moduleColors==module

datExpr <- as.data.frame(datExpr)

dd_2 <- datExpr[,moduleGenes]

dd_2 <- dd_2 %>%

rownames_to_column("ID")

dd <- dd_2 %>%

column_to_rownames("ID")

library(dplyr)

library(tibble)

library(tidyr)

test <- dd[1:10,1:10]

gene <- colnames(dd)

dd <- dd %>%

rownames_to_column("sample") %>%

mutate(group = datTraits$condition) %>%

dplyr::select(group,everything()) %>%

pivot_longer(cols = 3:ncol(.),

names_to = "gene",

values_to = "expression")

design

mylove = as.data.frame(design$high)

names(mylove) = "mylove"

modNames = substring(names(MEs), 3)

geneModuleMembership = as.data.frame(cor(datExpr, MEs, use = "p"))

MMPvalue = as.data.frame(corPvalueStudent(as.matrix(geneModuleMembership), nSamples))

names(geneModuleMembership) = paste("MM", modNames, sep="")

names(MMPvalue) = paste("p.MM", modNames, sep="")

geneTraitSignificance = as.data.frame(cor(datExpr, mylove$mylove, use = "p"))

GSPvalue = as.data.frame(corPvalueStudent(as.matrix(geneTraitSignificance), nSamples))

names(geneTraitSignificance) = paste("GS.", names(mylove), sep="")

names(GSPvalue) = paste("p.GS.", names(mylove), sep="");

module = "yellow"

column = match(module, modNames)

table(moduleColors)

moduleGenes = moduleColors==module

sizeGrWindow(7, 7)

par(mfrow = c(1,1))

MM <- geneModuleMembership[moduleGenes, column]

GS <- geneTraitSignificance[moduleGenes, 1]

verboseScatterplot(MM,GS,

xlab = paste("Module Membership in", module, "module"),

ylab = "Gene significance for body weight",

main = paste("Module membership vs. gene significance\n"),

cex.main = 1.2, cex.lab = 1.2, cex.axis = 1.2, col = module)

MM <- geneModuleMembership[moduleGenes, column]

MMP <- MMPvalue[moduleGenes, column]

GS <- geneTraitSignificance[moduleGenes, 1]

GSP <- GSPvalue[moduleGenes, 1]

# mydata_yellow <- data.frame(moduleGenes=colnames(datExpr)[moduleGenes],MM,MMP,GS,GSP)

# mydata_black <- data.frame(moduleGenes=colnames(datExpr)[moduleGenes],MM,MMP,GS,GSP)

# mydata_greenyellow <- data.frame(moduleGenes=colnames(datExpr)[moduleGenes],MM,MMP,GS,GSP)

# mydata_darkgreen <- data.frame(moduleGenes=colnames(datExpr)[moduleGenes],MM,MMP,GS,GSP)

# mydata_tan <- data.frame(moduleGenes=colnames(datExpr)[moduleGenes],MM,MMP,GS,GSP)

# mydata_green <- data.frame(moduleGenes=colnames(datExpr)[moduleGenes],MM,MMP,GS,GSP)

# mydata <- rbind(mydata_yellow)

gene <- as.character(gene)

gene <- gene

kegg <- read.gmt("resource/c2.cp.kegg.v2023.1.Hs.symbols.gmt")

y <- enricher(gene,TERM2GENE =kegg)

test <- as.data.frame(y)

dotplot(y)

gene <- gene

hallmark <- read.gmt("resource/h.all.v2023.1.Hs.symbols.gmt")

y <- enricher(gene,TERM2GENE =hallmark)

test <- as.data.frame(y)

dotplot(y)

gene = bitr(gene,

fromType="SYMBOL",

toType="ENTREZID",

OrgDb="org.Hs.eg.db")

go <- enrichGO(gene = gene$ENTREZID, OrgDb = "org.Hs.eg.db", ont="all")

library(ggplot2)

p <- barplot(go, label_format = 60,split="ONTOLOGY") +

facet_grid(ONTOLOGY~., scale="free")

p

rm(list = ls())

library(tidyr)

library(dplyr)

library(tibble)

library(survival)

library(caret)

library("glmnet")

library(survminer)

options(stringsAsFactors = F)

library(readxl)

rt1 <- read_excel(path = "resource/DTP_NCI60_ZSCORE.xlsx", skip = 7)

colnames(rt1) <- rt1[1,]

rt1 <- rt1[-1,-c(67,68)]

table(rt1$`FDA status`)

rt1 <- rt1[rt1$`FDA status` %in% c("FDA approved", "Clinical trial"),]

rt1 <- rt1[,-c(1, 3:6)]

write.table(rt1, file = "output/drug.txt",sep = "\t",row.names = F,quote = F)

rt2 <- read_excel(path = "resource/RNA__RNA_seq_composite_expression.xls", skip = 9)

colnames(rt2) <- rt2[1,]

rt2 <- rt2[-1,-c(2:6)]

write.table(rt2, file = "output/geneExp.txt",sep = "\t",row.names = F,quote = F)

rm(list = ls())

library(impute)

library(limma)

rt <- read.table("output/drug.txt",sep="\t",header=T,check.names=F)

rt <- as.matrix(rt)

rownames(rt) <- rt[,1]

drug <- rt[,2:ncol(rt)]

dimnames <- list(rownames(drug),colnames(drug))

data <- matrix(as.numeric(as.matrix(drug)),nrow=nrow(drug),dimnames=dimnames)

mat <- impute.knn(data)

drug <- mat$data

drug <- avereps(drug)

exp <- read.table("output/geneExp.txt", sep="\t", header=T, row.names = 1, check.names=F)

dim(exp)

exp[1:4, 1:4]

exp <- exp["C16orf74",]

outTab <- data.frame()

for(Gene in row.names(exp)){

x <- as.numeric(exp[Gene,])

for(Drug in row.names(drug)){

y <- as.numeric(drug[Drug,])

if (sd(y)!=0){

corT <- cor.test(x,y,method="spearman")

cor <- corT$estimate

pvalue <- corT$p.value

if(pvalue < 0.05){

outVector <- cbind(Gene,Drug,cor,pvalue)

outTab <- rbind(outTab,outVector)

}

}

}

}

outTab <- outTab[order(as.numeric(as.vector(outTab$pvalue))),]

write.table(outTab, file="output/drugCor.txt", sep="\t", row.names=F, quote=F)

library(ggplot2)

library(ggpubr)

plotList_1 <- list()

outTab$cor <- as.numeric(outTab$cor)

outTab$pvalue <- as.numeric(outTab$pvalue)

outTab <- outTab %>%

arrange(cor)

corPlotNum <- 16

for(i in 1:corPlotNum){

Gene <- outTab[i,1]

Drug <- outTab[i,2]

x <- as.numeric(exp[Gene,])

y <- as.numeric(drug[Drug,])

cor <- sprintf("%.03f",as.numeric(outTab[i,3]))

pvalue=0

if(as.numeric(outTab[i,4])<0.001){

pvalue="p<0.001"

}else{

pvalue=paste0("p=",sprintf("%.03f",as.numeric(outTab[i,4])))

}

df1 <- as.data.frame(cbind(x,y))

p1=ggplot(data = df1, aes(x = y, y = x))+

geom_point(size=1)+

stat_smooth(method="lm", se = FALSE, formula = y ~ x)+

labs(x = "IC50", y = "Expression", title = paste0(Gene, ", ", Drug), subtitle = paste0("Cor=", cor, ", ", pvalue))+

theme(axis.ticks = element_blank(), axis.text.y = element_blank(), axis.text.x = element_blank())+

theme_bw()

plotList_1[[i]]=p1

}

library(ggpubr)

ggarrange(plotlist = plotList_1, nrow = 4, ncol = 4)

plotList_2<- list()

corPlotNum<- 16

if(nrow(outTab)<corPlotNum){corPlotNum=nrow(outTab)}

for(i in 1:corPlotNum){

Gene <- outTab[i,1]

Drug <- outTab[i,2]

x <- as.numeric(exp[Gene,])

y <- as.numeric(drug[Drug,])

df1 <- as.data.frame(cbind(x,y))

colnames(df1)[2] <- "IC50"

df1$group <- ifelse(df1$x > median(df1$x), "low", "high")

compaired <- list(c("low", "high"))

p1 <- ggboxplot(df1,

x = "group", y = "IC50",

fill = "group", palette = c("#2878b5", "#c82423"),

add = "jitter", size = 0.5,

xlab = paste0("The_expression_of_", Gene),

ylab = paste0("IC50_of_", Drug)) +

stat_compare_means(comparisons = compaired,

method = "wilcox.test", #

symnum.args=list(cutpoints = c(0, 0.001, 0.01, 0.05, 1),

symbols = c("***", "**", "*", "ns")))

plotList_2[[i]]=p1

}

nrow<- ceiling(sqrt(corPlotNum))

ncol<- ceiling(corPlotNum/nrow)

ggarrange(plotlist=plotList_1,nrow=nrow,ncol=ncol)

ggarrange(plotlist=plotList_2,nrow=nrow,ncol=ncol)

ggarrange(plotlist=plotList_2,nrow=4,ncol=4)

rm(list = ls())

library(tibble)

library(survival)

library(caret)

# remove.packages("parallelly")

# install.packages("parallelly")

library("glmnet")

library(survminer)

library("survivalROC")

library(dplyr)

load(file = "resource/IMvigor210CoreBiologies.Rdata")

exprSet <- exprSet %>%

rownames_to_column("id")

gene_id <- annoData[,1:2]

colnames(gene_id)[1] <- "id"

exprSet <- inner_join(gene_id,exprSet,by="id")

exprSet <- na.omit(exprSet)

exprSet <- exprSet %>%

dplyr::select(-id) %>%

mutate(rowmean = rowMeans(.[,-1])) %>%

arrange(desc(rowmean)) %>%

distinct(symbol,.keep_all = T) %>%

select(-rowmean) %>%

column_to_rownames("symbol") %>%

t() %>%

as.data.frame() %>%

rownames_to_column("id")

surv <- dplyr::select(phenoData,os,censOS)

surv <- surv %>%

rownames_to_column("id")

data <- inner_join(surv,exprSet,by="id")

metadata <- dplyr::select(data,id,censOS)

colnames(metadata)[2] <- "sample"

metadata$sample <- as.factor(metadata$sample)

exprSet <- exprSet %>%

column_to_rownames("id") %>%

t() %>%

as.data.frame()

library(DESeq2)

dds <-DESeqDataSetFromMatrix(countData=exprSet,

colData=metadata,

design=~1)

nrow(dds)

rownames(dds)

dds <- dds[rowMedians(counts(dds))>0,]

nrow(dds)

system.time(vsd <- vst(dds, blind = FALSE))

plotPCA(vsd, "sample")

data <- as.data.frame(assay(vsd))

data <- data %>%

t() %>%

as.data.frame() %>%

rownames_to_column("id")

data <- inner_join(surv,data,by="id")

colnames(data) <- gsub("-","_",colnames(data))

outside <- data %>%

column_to_rownames("id") %>%

as.data.frame()

colnames(outside)[1] <- "futime"

colnames(outside)[2] <- "fustat"

OS_C16orf74 <- outside %>%

rownames_to_column("id") %>%

dplyr::select(id,futime,fustat,C16orf74)

write.table(OS_C16orf74,file="output/IMvigor210.txt",sep="\t",quote=F,row.names=F)

rm(list = ls())

library(survminer)

library(survival)

rt=read.table("output/IMvigor210.txt",header=T,sep="\t")

load(file = "resource/IMvigor210CoreBiologies.Rdata")

result <- dplyr::select(phenoData,binaryResponse)

result <- na.omit(result)

result <- result %>%

rownames_to_column("ID")

index <- result$ID

rt <- rt %>%

filter(id %in% index)

median_value <- median(rt$C16orf74, na.rm = TRUE)

rt$risk <- ifelse(rt$C16orf74 <= median_value, "low", "high")

diff=survdiff(Surv(futime, fustat) ~risk,data = rt)

pValue=1-pchisq(diff$chisq,df=1)

pValue=signif(pValue,4)

pValue=format(pValue, scientific = TRUE)

fit <- survfit(Surv(futime, fustat) ~ risk, data = rt)

summary(fit) #

ggsurvplot(fit,

pval = TRUE, #

pval.method = TRUE,#

conf.int = TRUE,#

risk.table = F, #

risk.table.col = "strata", #

linetype = "strata", #

#surv.median.line = "hv", #

xlab = "Time in years", #

xlim = c(0,24), #

break.time.by = 2, #

size=1, #

ggtheme = theme_bw(),

palette = c("#2878b5","#c82423")) + ggtitle("IMvigor210 cohort")

rm(list = ls())

library(survminer)

library(survival)

rt=read.table("output/IMvigor210.txt",header=T,sep="\t")

load(file = "resource/IMvigor210CoreBiologies.Rdata")

result <- dplyr::select(phenoData,binaryResponse)

result <- na.omit(result)

result <- result %>%

rownames_to_column("ID")

index <- result$ID

rt <- rt %>%

filter(id %in% index) %>%

column_to_rownames("id")

median_value <- median(rt$C16orf74, na.rm = TRUE)

rt$C16orf74 <- ifelse(rt$C16orf74 <= median_value, "low", "high")

genes <- "C16orf74"

your.surv <- Surv(rt$futime, rt$fustat)

your.km.plot <- function(genes,data){

print(genes)

group <- rt[,genes] #

survival_dat <- data.frame(group = group)

group <- factor(group, levels = c("low", "high"))

fit <- survfit(your.surv ~ group)

sdf <- survdiff(your.surv ~ group,rho=0)

p.val <- 1 - pchisq(sdf$chisq, length(sdf$n)-1)

p.val

photo2 <- ggsurvplot(fit,data = survival_dat, #

legend.title = genes,#

legend.labs = c("low","high"),

#legend = "top",

pval = T, #

#pval.method = TRUE,#

conf.int = TRUE,#

risk.table = F, #

#risk.table.col = "strata", #

risk.table.y.text = F,#

xlab = "Time in years", #

xlim = c(0,max(rt$futime)+1), #

break.time.by = 2, #

size = 1.5, #

palette = c("#2878b5", "#c82423")#

)

photo2

}

##2.测试函数功能

your.km.plot("C16orf74",data = rt)

rm(list = ls())

library(tibble)

library(dplyr)

library(pheatmap)

library(data.table)

library(ggpubr)

gene=read.table("output/IMvigor210.txt",sep="\t",header=T,row.names=1,check.names=F)

gene <- gene %>%

rownames_to_column("ID")

gene <- dplyr::select(gene,ID,C16orf74)

load(file = "resource/IMvigor210CoreBiologies.Rdata")

result <- dplyr::select(phenoData,binaryResponse)

result <- na.omit(result)

result <- result %>%

rownames_to_column("ID")

data <- inner_join(result,gene,by="ID")

data$binaryResponse

# data$C16orf74 <- log2(data$C16orf74)

p <- ggboxplot(data, x = "binaryResponse", y = "C16orf74",

color = "binaryResponse", palette = c("#2878b5","#c82423"),

add = "jitter")

# Add p-value

p + stat_compare_means()

rm(list = ls())

library(tidyr)

library(dplyr)

library(tibble)

load(file = "output/exprSet_vst.Rdata")

exprSet_vst <- exprSet_vst %>%

column_to_rownames("ID") %>%

t() %>%

as.data.frame() %>%

rownames_to_column("TCGA_ID")

metadata <- exprSet_vst %>%

dplyr::select(TCGA_ID,C16orf74)

median_value <- median(metadata$C16orf74, na.rm = TRUE)

metadata$C16orf74 <- ifelse(metadata$C16orf74 <= median_value, "low", "high")

colnames(metadata) <- c("TCGA_ID","group")

data <- inner_join(metadata,exprSet_vst,by="TCGA_ID")

data$group <- as.factor(data$group)

table(data$group)

library(ggpubr)

ggboxplot(

data, x = "group", y = "RPGR",

color = "group", palette = c("#00AFBB", "#E7B800"),

add = "jitter"

)+

stat_compare_means(method = "wilcox.test")

my.wilcox = function(x){

dd <- wilcox.test(data[,x] ~ group, data = data)

lowposition <- grep("low",data$group)

highposition <- grep("high",data$group)

lowcol <- data[,x][lowposition]

highcol<- data[,x][highposition]

lowMean = mean(lowcol)

highMean = mean(highcol)

logFC=highMean-lowMean

data.frame(gene=x,

p.value=dd$p.value,

logFC

)

}

my.wilcox("RPGR")

lapplylist = lapply(colnames(data)[-c(1:2)],my.wilcox)

wilcox_data <- do.call(rbind,lapplylist)

save(wilcox_data,file = "output/wilcox_data_logfc.Rdata")

rm(list = ls())

library(clusterProfiler)

load(file = "output/wilcox_data_logfc.Rdata")

gene_df <- wilcox_data

geneList <- gene_df$logFC

names(geneList) = gene_df$gene

geneList = sort(geneList, decreasing = TRUE)

head(geneList)

hallmarks <- read.gmt("resource/h.all.v2023.1.Hs.symbols.gmt")

y <- GSEA(geneList,TERM2GENE =hallmarks)

yd <- as.data.frame(y)

library(ggplot2)

dotplot(y,showCategory=30,

split=".sign",

font.size = 8,

label_format = 60)+facet_grid(~.sign)

library(stringi)

library(ggplot2)

dotplot(y,showCategory=12,split=".sign")+

facet_grid(~.sign)+

scale_y_discrete(labels=function(x) stri_sub(x,10))

library(enrichplot)

pathway.id = "HALLMARK_ALLOGRAFT_REJECTION"

gseaplot2(y,

color = "blue",

geneSetID = pathway.id,

pvalue_table = T)

pathway.id = "HALLMARK_IL2_STAT5_SIGNALING"

gseaplot2(y,

color = "blue",

geneSetID = pathway.id,

pvalue_table = T)

pathway.id = "HALLMARK_COMPLEMENT"

gseaplot2(y,

color = "blue",

geneSetID = pathway.id,

pvalue_table = T)

pathway.id = "HALLMARK_INTERFERON_GAMMA_RESPONSE"

gseaplot2(y,

color = "blue",

geneSetID = pathway.id,

pvalue_table = T)

pathway.id = "HALLMARK_INFLAMMATORY_RESPONSE"

gseaplot2(y,

color = "blue",

geneSetID = pathway.id,

pvalue_table = T)

pathway.id = "HALLMARK_IL6_JAK_STAT3_SIGNALING"

gseaplot2(y,

color = "blue",

geneSetID = pathway.id,

pvalue_table = T)

rm(list = ls())

library(clusterProfiler)

library(org.Hs.eg.db)

library(stringr)

library(BiocGenerics)

library(clusterProfiler)

library(enrichplot)

library(future)

library(future.apply)

load(file = "output/wilcox_data_logfc.Rdata")

gene_df <- wilcox_data

geneList <- gene_df$logFC

names(geneList) = gene_df$gene

geneList = sort(geneList, decreasing = TRUE)

head(geneList)

kegg <- read.gmt("resource/c2.cp.kegg.v2023.1.Hs.symbols.gmt")

y <- GSEA(geneList,TERM2GENE =kegg)

yd <- as.data.frame(y)

library(ggplot2)

library(stringi)

dotplot(y,showCategory=30,

split=".sign",

font.size = 8,

label_format = 60)+facet_grid(~.sign)+

scale_y_discrete(labels=function(x) stri_sub(x,6))

go_bp <- read.gmt("resource/c5.go.bp.v2023.1.Hs.symbols.gmt")

g <- GSEA(geneList,TERM2GENE =go_bp)

dotplot(g,showCategory=10,

split=".sign",

font.size = 8,

label_format = 60)+facet_grid(~.sign)

go_cc <- read.gmt("resource/c5.go.cc.v2023.1.Hs.symbols.gmt")

g <- GSEA(geneList,TERM2GENE =go_cc)

dotplot(g,showCategory=10,

split=".sign",

font.size = 8,

label_format = 60)+facet_grid(~.sign)

go_mf <- read.gmt("resource/c5.go.mf.v2023.1.Hs.symbols.gmt")

g <- GSEA(geneList,TERM2GENE =go_mf)

dotplot(g,showCategory=10,

split=".sign",

font.size = 8,

label_format = 60)+facet_grid(~.sign)

rm(list = ls())

library(tidyr)

library(dplyr)

library(tibble)

library(export)

library(DESeq2)

library(ggpubr)

library(ggplot2)

library(survminer)

library(survival)

library(ggprism)

library(tidyverse)

library(timeROC)

library(data.table)

library(pheatmap)

library(rms)

library(foreign)

library(pec)

load(file = "output/exprSet_vst.Rdata")

exprSet_vst <- exprSet_vst %>%

column_to_rownames("ID") %>%

t() %>%

as.data.frame() %>%

rownames_to_column("TCGA_ID") %>%

dplyr::select(TCGA_ID,C16orf74)

cli=read.table("resource/HNSC_clinical.txt",header=T,sep="\t",check.names=F,row.names=1)

sur=read.table("resource/HNSC_survival.txt",header=T,sep="\t",check.names=F,row.names=1)

colnames(sur)[2] <- "fustat"

colnames(sur)[1] <- "futime"

clinical_new <- cli %>%

rownames_to_column("TCGA_ID")

survival_new <- sur %>%

rownames_to_column("TCGA_ID")

clinical_final <- merge(clinical_new,survival_new,by="TCGA_ID")

clinical_final <- merge(clinical_final,exprSet_vst,by="TCGA_ID")

clinical_final$age <- ifelse(clinical_final$age > 60, ">60", "<=60")

clinical <- clinical_final %>%

dplyr::select(TCGA_ID,fustat,age,gender,histologic_grade,

lymphovascular_invasion,perineural_invasion,cancer_status,primary_therapy_outcome,

pathologic_t,pathologic_n,pathologic_m,pathologic_stage,

clinical_t,clinical_n,clinical_m,clinical_stage,

C16orf74,futime) %>%

column_to_rownames("TCGA_ID")

rt <- clinical %>%

dplyr::select(C16orf74,everything())

rt$futime <- round(rt$futime/365,3) #

age <- rt[,c(1,2,3,18)]

age$age <- gsub("<=60", "age<=60", age$age)

age$age <- gsub(">60", "age>60", age$age)

age <- age %>%

filter(age == "age<=60") %>%

dplyr::select(-age)

data <- age %>%

dplyr::select(fustat,futime,everything())

colnames(data)[1:2] <- c("OS","OS.time")

res.cut <- surv_cutpoint(data, time = "OS.time",

event = "OS",

variables = names(data)[3:ncol(data)],

minprop = 0.3) #

res.cat <- surv_categorize(res.cut)

summary(res.cut)

str(res.cat)

genes <- "C16orf74"

your.surv <- Surv(res.cat$OS.time, res.cat$OS)

your.km.plot <- function(genes,data){

print(genes)

group <- res.cat[,genes]

survival_dat <- data.frame(group = group)

group <- factor(group, levels = c("low", "high"))

fit <- survfit(your.surv ~ group)

sdf <- survdiff(your.surv ~ group,rho=0)

p.val <- 1 - pchisq(sdf$chisq, length(sdf$n)-1)

p.val

photo2 <- ggsurvplot(fit,data = survival_dat, #

legend.title = genes,#

legend.labs = c("low","high"),

pval = T, #

conf.int = TRUE,

risk.table = F, #

#risk.table.col = "strata", #

risk.table.y.text = F,#

xlab = "Time in years",

xlim = c(0,max(res.cat$OS.time)+1), #

break.time.by = 2,

size = 1.5, #

#ggtheme = theme_bw(), #

palette = c("#2878b5", "#c82423")#

) + ggtitle("Age<=60")

photo2

photo2$table <- photo2$table

photo2

}

##2.

your.km.plot("C16orf74")

age <- rt[,c(1,2,3,18)]

age$age <- gsub("<=60", "age<=60", age$age)

age$age <- gsub(">60", "age>60", age$age)

age <- age %>%

filter(age == "age>60") %>%

dplyr::select(-age)

data <- age %>%

dplyr::select(fustat,futime,everything())

colnames(data)[1:2] <- c("OS","OS.time")

res.cut <- surv_cutpoint(data, time = "OS.time",

event = "OS",

variables = names(data)[3:ncol(data)],

minprop = 0.3) #

res.cat <- surv_categorize(res.cut)

summary(res.cut)

str(res.cat)

genes <- "C16orf74"

your.surv <- Surv(res.cat$OS.time, res.cat$OS)

your.km.plot <- function(genes,data){

print(genes)

group <- res.cat[,genes]

survival_dat <- data.frame(group = group)

group <- factor(group, levels = c("low", "high"))

fit <- survfit(your.surv ~ group)

sdf <- survdiff(your.surv ~ group,rho=0)

p.val <- 1 - pchisq(sdf$chisq, length(sdf$n)-1)

p.val

photo2 <- ggsurvplot(fit,data = survival_dat,

legend.title = genes,#

legend.labs = c("low","high"),

#legend = "top",#

pval = T, #

#pval.method = TRUE,#

conf.int = TRUE,#

risk.table = F, #

#risk.table.col = "strata", #

risk.table.y.text = F,#

xlab = "Time in years",

xlim = c(0,max(res.cat$OS.time)+1), #

break.time.by = 2, #

size = 1.5, #

#ggtheme = theme_bw(), #

palette = c("#2878b5", "#c82423")#

) + ggtitle("Age>60")

photo2

photo2$table <- photo2$table

}

##2.

your.km.plot("C16orf74")

gender <- rt[,c(1,2,4,18)]

gender <- na.omit(gender)

female <- gender[gender$gender == "female",]

data <- female %>%

dplyr::select(-gender) %>%

dplyr::select(fustat,futime,everything())

colnames(data)[1:2] <- c("OS","OS.time")

res.cut <- surv_cutpoint(data, time = "OS.time",

event = "OS",

variables = names(data)[3:ncol(data)],

minprop = 0.3) #

res.cat <- surv_categorize(res.cut)

summary(res.cut)

str(res.cat)

genes <- "C16orf74"

your.surv <- Surv(res.cat$OS.time, res.cat$OS)

your.km.plot <- function(genes,data){

print(genes)

group <- res.cat[,genes] #

survival_dat <- data.frame(group = group)

group <- factor(group, levels = c("low", "high"))

fit <- survfit(your.surv ~ group)

sdf <- survdiff(your.surv ~ group,rho=0)

p.val <- 1 - pchisq(sdf$chisq, length(sdf$n)-1)

p.val

photo2 <- ggsurvplot(fit,data = survival_dat, #

legend.title = genes,#

legend.labs = c("low","high"), #

#legend = "top",#

pval = T, #

#pval.method = TRUE,#

conf.int = TRUE,#

risk.table = F, #

#risk.table.col = "strata", #

risk.table.y.text = F,#

#linetype = "strata", #

#surv.median.line = "hv", #

xlab = "Time in years", #

xlim = c(0,max(res.cat$OS.time)+1), #

break.time.by = 2, #

size = 1.5, #

#ggtheme = theme_bw(), #

palette = c("#2878b5", "#c82423")#

) + ggtitle("female")

photo2 #

photo2$table <- photo2$table

}

##2.

your.km.plot("C16orf74")

gender <- rt[,c(1,2,4,18)]

gender <- na.omit(gender)

male <- gender[gender$gender == "male",]

data <- male %>%

dplyr::select(-gender) %>%

dplyr::select(fustat,futime,everything())

colnames(data)[1:2] <- c("OS","OS.time")

res.cut <- surv_cutpoint(data, time = "OS.time",

event = "OS",

variables = names(data)[3:ncol(data)],

minprop = 0.3) #

res.cat <- surv_categorize(res.cut)

summary(res.cut)

str(res.cat)

genes <- "C16orf74"

your.surv <- Surv(res.cat$OS.time, res.cat$OS)

your.km.plot <- function(genes,data){

print(genes)

group <- res.cat[,genes] #

survival_dat <- data.frame(group = group)

group <- factor(group, levels = c("low", "high"))

fit <- survfit(your.surv ~ group)

sdf <- survdiff(your.surv ~ group,rho=0)

p.val <- 1 - pchisq(sdf$chisq, length(sdf$n)-1)

p.val

photo2 <- ggsurvplot(fit,data = survival_dat, #

legend.title = genes,#

legend.labs = c("low","high"), #

#legend = "top",#

pval = T, #

#pval.method = TRUE,#

conf.int = TRUE,#

risk.table = F, #

#risk.table.col = "strata", #

risk.table.y.text = F,#

#linetype = "strata", #

#surv.median.line = "hv", #

xlab = "Time in years", #

xlim = c(0,max(res.cat$OS.time)+1), #

break.time.by = 2, #

size = 1.5, #

#ggtheme = theme_bw(),

palette = c("#2878b5", "#c82423")#

) + ggtitle("male")

photo2 #

photo2$table <- photo2$table

}

##2.

your.km.plot("C16orf74")

lymphovascular_invasion <- rt[,c(1,2,6,18)]

lymphovascular_invasion <- na.omit(lymphovascular_invasion)

NO <- lymphovascular_invasion[lymphovascular_invasion$lymphovascular_invasion == "NO",]

data <- NO %>%

dplyr::select(-lymphovascular_invasion) %>%

dplyr::select(fustat,futime,everything())

colnames(data)[1:2] <- c("OS","OS.time")

res.cut <- surv_cutpoint(data, time = "OS.time",

event = "OS",

variables = names(data)[3:ncol(data)],

minprop = 0.3) #

res.cat <- surv_categorize(res.cut)

summary(res.cut)

str(res.cat)

genes <- "C16orf74"

your.surv <- Surv(res.cat$OS.time, res.cat$OS)

your.km.plot <- function(genes,data){

print(genes)

group <- res.cat[,genes]

survival_dat <- data.frame(group = group)

group <- factor(group, levels = c("low", "high"))

fit <- survfit(your.surv ~ group)

sdf <- survdiff(your.surv ~ group,rho=0)

p.val <- 1 - pchisq(sdf$chisq, length(sdf$n)-1)

p.val

photo2 <- ggsurvplot(fit,data = survival_dat, #

legend.title = genes,#

legend.labs = c("low","high"), #

#legend = "top",

pval = T, #

#pval.method = TRUE,#

conf.int = TRUE,#

risk.table = F, #

#risk.table.col = "strata", #

risk.table.y.text = F,#

#linetype = "strata", #

#surv.median.line = "hv", #

xlab = "Time in years", #

xlim = c(0,max(res.cat$OS.time)+1), #

break.time.by = 2, #

size = 1.5, #

#ggtheme = theme_bw(), #

palette = c("#2878b5", "#c82423")#

) + ggtitle("lymphovascular_invasion_NO")

photo2

photo2$table <- photo2$table

}

##2

your.km.plot("C16orf74")

YES <- lymphovascular_invasion[lymphovascular_invasion$lymphovascular_invasion == "YES",]

data <- YES %>%

dplyr::select(-lymphovascular_invasion) %>%

dplyr::select(fustat,futime,everything())

colnames(data)[1:2] <- c("OS","OS.time")

res.cut <- surv_cutpoint(data, time = "OS.time",

event = "OS",

variables = names(data)[3:ncol(data)],

minprop = 0.3) #

res.cat <- surv_categorize(res.cut)

summary(res.cut)

str(res.cat)

###

genes <- "C16orf74"

your.surv <- Surv(res.cat$OS.time, res.cat$OS)

your.km.plot <- function(genes,data){

print(genes)

group <- res.cat[,genes] #

survival_dat <- data.frame(group = group)

group <- factor(group, levels = c("low", "high"))

fit <- survfit(your.surv ~ group)

sdf <- survdiff(your.surv ~ group,rho=0)

p.val <- 1 - pchisq(sdf$chisq, length(sdf$n)-1)

p.val

photo2 <- ggsurvplot(fit,data = survival_dat, #

legend.title = genes,

legend.labs = c("low","high"), #

#legend = "top",#

pval = T, #

#pval.method = TRUE,#

conf.int = TRUE,#

risk.table = F, #

#risk.table.col = "strata", #

risk.table.y.text = F,#组

#linetype = "strata", #

xlab = "Time in years", #

xlim = c(0,max(res.cat$OS.time)+1), #

break.time.by = 2, #

size = 1.5, #

#ggtheme = theme_bw(), #

palette = c("#2878b5", "#c82423")#

) + ggtitle("lymphovascular_invasion_YES")

photo2

photo2$table <- photo2$table

}

##2.

your.km.plot("C16orf74")

perineural_invasion <- rt[,c(1,2,7,18)]

perineural_invasion <- na.omit(perineural_invasion)

NO <- perineural_invasion[perineural_invasion$perineural_invasion == "NO",]

data <- NO %>%

dplyr::select(-perineural_invasion) %>%

dplyr::select(fustat,futime,everything())

colnames(data)[1:2] <- c("OS","OS.time")

res.cut <- surv_cutpoint(data, time = "OS.time",

event = "OS",

variables = names(data)[3:ncol(data)],

minprop = 0.3) #

res.cat <- surv_categorize(res.cut)

summary(res.cut)

str(res.cat)

genes <- "C16orf74"

your.surv <- Surv(res.cat$OS.time, res.cat$OS)

your.km.plot <- function(genes,data){

print(genes)

group <- res.cat[,genes]

survival_dat <- data.frame(group = group)

group <- factor(group, levels = c("low", "high"))

fit <- survfit(your.surv ~ group)

sdf <- survdiff(your.surv ~ group,rho=0)

p.val <- 1 - pchisq(sdf$chisq, length(sdf$n)-1)

p.val

photo2 <- ggsurvplot(fit,data = survival_dat, #

legend.title = genes,#

legend.labs = c("low","high"), #

#legend = "top",#

pval = T, #

#pval.method = TRUE,#

conf.int = TRUE,#

risk.table = F, #

#risk.table.col = "strata", #

risk.table.y.text = F,#

xlab = "Time in years", #

xlim = c(0,max(res.cat$OS.time)+1), #

break.time.by = 2, #

size = 1.5, #

#ggtheme = theme_bw(), #

palette = c("#2878b5", "#c82423")#

) + ggtitle("perineural_invasion_NO")

photo2 #

photo2$table <- photo2$table

}

##2.

your.km.plot("C16orf74")

YES <- perineural_invasion[perineural_invasion$perineural_invasion == "YES",]

data <- YES %>%

dplyr::select(-perineural_invasion) %>%

dplyr::select(fustat,futime,everything())

colnames(data)[1:2] <- c("OS","OS.time")

res.cut <- surv_cutpoint(data, time = "OS.time",

event = "OS",

variables = names(data)[3:ncol(data)],

minprop = 0.3) #

res.cat <- surv_categorize(res.cut)

summary(res.cut)

str(res.cat)

genes <- "C16orf74"

your.surv <- Surv(res.cat$OS.time, res.cat$OS)

your.km.plot <- function(genes,data){

print(genes)

group <- res.cat[,genes] #

survival_dat <- data.frame(group = group)

group <- factor(group, levels = c("low", "high"))

fit <- survfit(your.surv ~ group)

sdf <- survdiff(your.surv ~ group,rho=0)

p.val <- 1 - pchisq(sdf$chisq, length(sdf$n)-1)

p.val

photo2 <- ggsurvplot(fit,data = survival_dat, #

legend.title = genes,#

legend.labs = c("low","high"), #

#legend = "top",#

pval = T, #

#pval.method = TRUE,#

conf.int = TRUE,#

risk.table = F, #

#risk.table.col = "strata", #

risk.table.y.text = F,#

#linetype = "strata", #

#surv.median.line = "hv", #

xlab = "Time in years", #x

xlim = c(0,max(res.cat$OS.time)+1), #

break.time.by = 2, #

size = 1.5, #

#ggtheme = theme_bw(), #

palette = c("#2878b5", "#c82423")#

) + ggtitle("perineural_invasion_YES")

photo2

photo2$table <- photo2$table

}

##2.

your.km.plot("C16orf74")

cancer_status <- rt[,c(1,2,8,18)]

cancer_status <- na.omit(cancer_status)

TUMOR_FREE <- cancer_status[cancer_status$cancer_status == "TUMOR FREE",]

data <- TUMOR_FREE %>%

dplyr::select(-cancer_status) %>%

dplyr::select(fustat,futime,everything())

colnames(data)[1:2] <- c("OS","OS.time")

res.cut <- surv_cutpoint(data, time = "OS.time",

event = "OS",

variables = names(data)[3:ncol(data)],

minprop = 0.3) #

res.cat <- surv_categorize(res.cut)

summary(res.cut)

str(res.cat)

genes <- "C16orf74"

your.surv <- Surv(res.cat$OS.time, res.cat$OS)

your.km.plot <- function(genes,data){

print(genes)

group <- res.cat[,genes]

survival_dat <- data.frame(group = group)

group <- factor(group, levels = c("low", "high"))

fit <- survfit(your.surv ~ group)

sdf <- survdiff(your.surv ~ group,rho=0)

p.val <- 1 - pchisq(sdf$chisq, length(sdf$n)-1)

p.val

photo2 <- ggsurvplot(fit,data = survival_dat,

legend.title = genes,#

legend.labs = c("low","high"), #

#legend = "top",#

pval = T, #

#pval.method = TRUE,#

conf.int = TRUE,#

risk.table = F, #

#risk.table.col = "strata",

risk.table.y.text = F,

#linetype = "strata", #

#surv.median.line = "hv", #

xlab = "Time in years", #

xlim = c(0,max(res.cat$OS.time)+1),

break.time.by = 2, #

size = 1.5,

#ggtheme = theme_bw(),

palette = c("#2878b5", "#c82423")#

) + ggtitle("TUMOR_FREE")

photo2 #

#

#

photo2$table <- photo2$table

# Changing the font size, style and color of photo2

# survival curves, risk table

photo2 #

}

##2.

your.km.plot("C16orf74")

WITH_TUMOR <- cancer_status[cancer_status$cancer_status == "WITH TUMOR",]

data <- WITH_TUMOR %>%

dplyr::select(-cancer_status) %>%

dplyr::select(fustat,futime,everything())

colnames(data)[1:2] <- c("OS","OS.time")

res.cut <- surv_cutpoint(data, time = "OS.time",

event = "OS",

variables = names(data)[3:ncol(data)],

minprop = 0.3) #

res.cat <- surv_categorize(res.cut)

summary(res.cut)

str(res.cat)

genes <- "C16orf74"

your.surv <- Surv(res.cat$OS.time, res.cat$OS)

your.km.plot <- function(genes,data){

print(genes)

group <- res.cat[,genes]

survival_dat <- data.frame(group = group)

group <- factor(group, levels = c("low", "high"))

fit <- survfit(your.surv ~ group)

sdf <- survdiff(your.surv ~ group,rho=0)

p.val <- 1 - pchisq(sdf$chisq, length(sdf$n)-1)

p.val

photo2 <- ggsurvplot(fit,data = survival_dat,

legend.title = genes,#

legend.labs = c("low","high"), #

#legend = "top",#

pval = T, #

#pval.method = TRUE,#

conf.int = TRUE,#

risk.table = F, #

#risk.table.col = "strata",

risk.table.y.text = F,

#linetype = "strata", #

#surv.median.line = "hv", #

xlab = "Time in years", #

xlim = c(0,max(res.cat$OS.time)+1),

break.time.by = 2, #

size = 1.5,

#ggtheme = theme_bw(),

palette = c("#2878b5", "#c82423")#

) + ggtitle("WITH_TUMOR")

photo2 #

#

#

photo2$table <- photo2$table

# Changing the font size, style and color of photo2

# survival curves, risk table

photo2 #

}

##2.

your.km.plot("C16orf74")

pathologic_t <- rt[,c(1,2,10,18)]

pathologic_t$pathologic_t <- gsub("T1", "T1/2", pathologic_t$pathologic_t)

pathologic_t$pathologic_t <- gsub("T2", "T1/2", pathologic_t$pathologic_t)

pathologic_t$pathologic_t <- gsub("T3", "T3/4", pathologic_t$pathologic_t)

pathologic_t$pathologic_t <- gsub("T4", "T3/4", pathologic_t$pathologic_t)

pathologic_t <- na.omit(pathologic_t)

T1_2 <- pathologic_t[pathologic_t$pathologic_t == "T1/2",]

data <- T1_2 %>%

dplyr::select(-pathologic_t) %>%

dplyr::select(fustat,futime,everything())

colnames(data)[1:2] <- c("OS","OS.time")

res.cut <- surv_cutpoint(data, time = "OS.time",

event = "OS",

variables = names(data)[3:ncol(data)],

minprop = 0.3) #

res.cat <- surv_categorize(res.cut)

summary(res.cut)

str(res.cat)

genes <- "C16orf74"

your.surv <- Surv(res.cat$OS.time, res.cat$OS)

your.km.plot <- function(genes,data){

print(genes)

group <- res.cat[,genes]

survival_dat <- data.frame(group = group)

group <- factor(group, levels = c("low", "high"))

fit <- survfit(your.surv ~ group)

sdf <- survdiff(your.surv ~ group,rho=0)

p.val <- 1 - pchisq(sdf$chisq, length(sdf$n)-1)

p.val

photo2 <- ggsurvplot(fit,data = survival_dat,

legend.title = genes,#

legend.labs = c("low","high"), #

#legend = "top",#

pval = T, #

#pval.method = TRUE,#

conf.int = TRUE,#

risk.table = F, #

#risk.table.col = "strata",

risk.table.y.text = F,

#linetype = "strata", #

#surv.median.line = "hv", #

xlab = "Time in years", #

xlim = c(0,max(res.cat$OS.time)+1),

break.time.by = 2, #

size = 1.5,

#ggtheme = theme_bw(),

palette = c("#2878b5", "#c82423")#

) + ggtitle("pathologic_T1_2")

photo2 #

#

#

photo2$table <- photo2$table

# Changing the font size, style and color of photo2

# survival curves, risk table

photo2 #

}

##2.

your.km.plot("C16orf74")

T3_4 <- pathologic_t[pathologic_t$pathologic_t == "T3/4",]

data <- T3_4 %>%

dplyr::select(-pathologic_t) %>%

dplyr::select(fustat,futime,everything())

colnames(data)[1:2] <- c("OS","OS.time")

res.cut <- surv_cutpoint(data, time = "OS.time",

event = "OS",

variables = names(data)[3:ncol(data)],

minprop = 0.3) #

res.cat <- surv_categorize(res.cut)

summary(res.cut)

str(res.cat)

genes <- "C16orf74"

your.surv <- Surv(res.cat$OS.time, res.cat$OS)

your.km.plot <- function(genes,data){

print(genes)

group <- res.cat[,genes]

survival_dat <- data.frame(group = group)

group <- factor(group, levels = c("low", "high"))

fit <- survfit(your.surv ~ group)

sdf <- survdiff(your.surv ~ group,rho=0)

p.val <- 1 - pchisq(sdf$chisq, length(sdf$n)-1)

p.val

photo2 <- ggsurvplot(fit,data = survival_dat,

legend.title = genes,#

legend.labs = c("low","high"), #

#legend = "top",#

pval = T, #

#pval.method = TRUE,#

conf.int = TRUE,#

risk.table = F, #

#risk.table.col = "strata",

risk.table.y.text = F,

#linetype = "strata", #

#surv.median.line = "hv", #

xlab = "Time in years", #

xlim = c(0,max(res.cat$OS.time)+1),

break.time.by = 2, #

size = 1.5,

#ggtheme = theme_bw(),

palette = c("#2878b5", "#c82423")#

) + ggtitle("pathologic_T3_4")

photo2 #

#

#

photo2$table <- photo2$table

# Changing the font size, style and color of photo2

# survival curves, risk table

photo2 #

}

##2.

your.km.plot("C16orf74")

clinical_t <- rt[,c(1,2,14,18)]

clinical_t$clinical_t <- gsub("T1", "T1/2", clinical_t$clinical_t)

clinical_t$clinical_t <- gsub("T2", "T1/2", clinical_t$clinical_t)

clinical_t$clinical_t <- gsub("T3", "T3/4", clinical_t$clinical_t)

clinical_t$clinical_t <- gsub("T4", "T3/4", clinical_t$clinical_t)

clinical_t <- na.omit(clinical_t)

T1_2 <- clinical_t[clinical_t$clinical_t == "T1/2",]

data <- T1_2 %>%

dplyr::select(-clinical_t) %>%

dplyr::select(fustat,futime,everything())

colnames(data)[1:2] <- c("OS","OS.time")

res.cut <- surv_cutpoint(data, time = "OS.time",

event = "OS",

variables = names(data)[3:ncol(data)],

minprop = 0.3) #

res.cat <- surv_categorize(res.cut)

summary(res.cut)

str(res.cat)

genes <- "C16orf74"

your.surv <- Surv(res.cat$OS.time, res.cat$OS)

your.km.plot <- function(genes,data){

print(genes)

group <- res.cat[,genes]

survival_dat <- data.frame(group = group)

group <- factor(group, levels = c("low", "high"))

fit <- survfit(your.surv ~ group)

sdf <- survdiff(your.surv ~ group,rho=0)

p.val <- 1 - pchisq(sdf$chisq, length(sdf$n)-1)

p.val

photo2 <- ggsurvplot(fit,data = survival_dat,

legend.title = genes,#

legend.labs = c("low","high"), #

#legend = "top",#

pval = T, #

#pval.method = TRUE,#

conf.int = TRUE,#

risk.table = F, #

#risk.table.col = "strata",

risk.table.y.text = F,

#linetype = "strata", #

#surv.median.line = "hv", #

xlab = "Time in years", #

xlim = c(0,max(res.cat$OS.time)+1),

break.time.by = 2, #

size = 1.5,

#ggtheme = theme_bw(),

palette = c("#2878b5", "#c82423")#

) + ggtitle("clinical_T_1_2")

photo2 #

#

#

photo2$table <- photo2$table

# Changing the font size, style and color of photo2

# survival curves, risk table

photo2 #

}

##2.

your.km.plot("C16orf74")

T3_4 <- clinical_t[clinical_t$clinical_t == "T3/4",]

data <- T3_4 %>%

dplyr::select(-clinical_t) %>%

dplyr::select(fustat,futime,everything())

colnames(data)[1:2] <- c("OS","OS.time")

res.cut <- surv_cutpoint(data, time = "OS.time",

event = "OS",

variables = names(data)[3:ncol(data)],

minprop = 0.3) #

res.cat <- surv_categorize(res.cut)

summary(res.cut)

str(res.cat)

genes <- "C16orf74"

your.surv <- Surv(res.cat$OS.time, res.cat$OS)

your.km.plot <- function(genes,data){

print(genes)

group <- res.cat[,genes]

survival_dat <- data.frame(group = group)

group <- factor(group, levels = c("low", "high"))

fit <- survfit(your.surv ~ group)

sdf <- survdiff(your.surv ~ group,rho=0)

p.val <- 1 - pchisq(sdf$chisq, length(sdf$n)-1)

p.val

photo2 <- ggsurvplot(fit,data = survival_dat,

legend.title = genes,#

legend.labs = c("low","high"), #

#legend = "top",#

pval = T, #

#pval.method = TRUE,#

conf.int = TRUE,#

risk.table = F, #

#risk.table.col = "strata",

risk.table.y.text = F,

#linetype = "strata", #

#surv.median.line = "hv", #

xlab = "Time in years", #

xlim = c(0,max(res.cat$OS.time)+1),

break.time.by = 2, #

size = 1.5,

#ggtheme = theme_bw(),

palette = c("#2878b5", "#c82423")#

) + ggtitle("clinical_T_3_4")

photo2 #

#

#

photo2$table <- photo2$table

# Changing the font size, style and color of photo2

# survival curves, risk table

photo2 #

}

##2.

your.km.plot("C16orf74")

pathologic_stage <- rt[,c(1,2,13,18)]

pathologic_stage$pathologic_stage <- gsub(" ", "", pathologic_stage$pathologic_stage)

pathologic_stage$pathologic_stage <- gsub("IV", "4", pathologic_stage$pathologic_stage)

pathologic_stage$pathologic_stage <- gsub("III", "3", pathologic_stage$pathologic_stage)

pathologic_stage$pathologic_stage <- gsub("II", "2", pathologic_stage$pathologic_stage)

pathologic_stage$pathologic_stage <- gsub("I", "1", pathologic_stage$pathologic_stage)

pathologic_stage$pathologic_stage <- gsub("1", "I+II", pathologic_stage$pathologic_stage)

pathologic_stage$pathologic_stage <- gsub("2", "I+II", pathologic_stage$pathologic_stage)

pathologic_stage$pathologic_stage <- gsub("3", "III+IV", pathologic_stage$pathologic_stage)

pathologic_stage$pathologic_stage <- gsub("4", "III+IV", pathologic_stage$pathologic_stage)

I_II <- pathologic_stage[pathologic_stage$pathologic_stage == "I+II",]

data <- I_II %>%

dplyr::select(-pathologic_stage) %>%

dplyr::select(fustat,futime,everything())

colnames(data)[1:2] <- c("OS","OS.time")

res.cut <- surv_cutpoint(data, time = "OS.time",

event = "OS",

variables = names(data)[3:ncol(data)],

minprop = 0.3) #

res.cat <- surv_categorize(res.cut)

summary(res.cut)

str(res.cat)

genes <- "C16orf74"

your.surv <- Surv(res.cat$OS.time, res.cat$OS)

your.km.plot <- function(genes,data){

print(genes)

group <- res.cat[,genes]

survival_dat <- data.frame(group = group)

group <- factor(group, levels = c("low", "high"))

fit <- survfit(your.surv ~ group)

sdf <- survdiff(your.surv ~ group,rho=0)

p.val <- 1 - pchisq(sdf$chisq, length(sdf$n)-1)

p.val

photo2 <- ggsurvplot(fit,data = survival_dat,

legend.title = genes,#

legend.labs = c("low","high"), #

#legend = "top",#

pval = T, #

#pval.method = TRUE,#

conf.int = TRUE,#

risk.table = F, #

#risk.table.col = "strata",

risk.table.y.text = F,

#linetype = "strata", #

#surv.median.line = "hv", #

xlab = "Time in years", #

xlim = c(0,max(res.cat$OS.time)+1),

break.time.by = 2, #

size = 1.5,

#ggtheme = theme_bw(),

palette = c("#2878b5", "#c82423")#

) + ggtitle("pathologic_stage_I+II")

photo2 #

#

#

photo2$table <- photo2$table

# Changing the font size, style and color of photo2

# survival curves, risk table

photo2 #

}

##2.

your.km.plot("C16orf74")

III_IV <- pathologic_stage[pathologic_stage$pathologic_stage == "III+IV",]

data <- III_IV %>%

dplyr::select(-pathologic_stage) %>%

dplyr::select(fustat,futime,everything())

colnames(data)[1:2] <- c("OS","OS.time")

res.cut <- surv_cutpoint(data, time = "OS.time",

event = "OS",

variables = names(data)[3:ncol(data)],

minprop = 0.3) #

res.cat <- surv_categorize(res.cut)

summary(res.cut)

str(res.cat)

genes <- "C16orf74"

your.surv <- Surv(res.cat$OS.time, res.cat$OS)

your.km.plot <- function(genes,data){

print(genes)

group <- res.cat[,genes]

survival_dat <- data.frame(group = group)

group <- factor(group, levels = c("low", "high"))

fit <- survfit(your.surv ~ group)

sdf <- survdiff(your.surv ~ group,rho=0)

p.val <- 1 - pchisq(sdf$chisq, length(sdf$n)-1)

p.val

photo2 <- ggsurvplot(fit,data = survival_dat,

legend.title = genes,#

legend.labs = c("low","high"), #

#legend = "top",#

pval = T, #

#pval.method = TRUE,#

conf.int = TRUE,#

risk.table = F, #

#risk.table.col = "strata",

risk.table.y.text = F,

#linetype = "strata", #

#surv.median.line = "hv", #

xlab = "Time in years", #

xlim = c(0,max(res.cat$OS.time)+1),

break.time.by = 2, #

size = 1.5,

#ggtheme = theme_bw(),

palette = c("#2878b5", "#c82423")#

) + ggtitle("pathologic_stage_III+IV")

photo2 #

#

#

photo2$table <- photo2$table

# Changing the font size, style and color of photo2

# survival curves, risk table

photo2 #

}

##2.

your.km.plot("C16orf74")

clinical_stage <- rt[,c(1,2,17,18)]

clinical_stage$clinical_stage <- gsub(" ", "", clinical_stage$clinical_stage)

clinical_stage$clinical_stage <- gsub("IV", "4", clinical_stage$clinical_stage)

clinical_stage$clinical_stage <- gsub("III", "3", clinical_stage$clinical_stage)

clinical_stage$clinical_stage <- gsub("II", "2", clinical_stage$clinical_stage)

clinical_stage$clinical_stage <- gsub("I", "1", clinical_stage$clinical_stage)

clinical_stage$clinical_stage <- gsub("1", "I+II", clinical_stage$clinical_stage)

clinical_stage$clinical_stage <- gsub("2", "I+II", clinical_stage$clinical_stage)

clinical_stage$clinical_stage <- gsub("3", "III+IV", clinical_stage$clinical_stage)

clinical_stage$clinical_stage <- gsub("4", "III+IV", clinical_stage$clinical_stage)

I_II <- clinical_stage[clinical_stage$clinical_stage == "I+II",]

data <- I_II %>%

dplyr::select(-clinical_stage) %>%

dplyr::select(fustat,futime,everything())

colnames(data)[1:2] <- c("OS","OS.time")

res.cut <- surv_cutpoint(data, time = "OS.time",

event = "OS",

variables = names(data)[3:ncol(data)],

minprop = 0.3) #

res.cat <- surv_categorize(res.cut)

summary(res.cut)

str(res.cat)

genes <- "C16orf74"

your.surv <- Surv(res.cat$OS.time, res.cat$OS)

your.km.plot <- function(genes,data){

print(genes)

group <- res.cat[,genes]

survival_dat <- data.frame(group = group)

group <- factor(group, levels = c("low", "high"))

fit <- survfit(your.surv ~ group)

sdf <- survdiff(your.surv ~ group,rho=0)

p.val <- 1 - pchisq(sdf$chisq, length(sdf$n)-1)

p.val

photo2 <- ggsurvplot(fit,data = survival_dat,

legend.title = genes,#

legend.labs = c("low","high"), #

#legend = "top",#

pval = T, #

#pval.method = TRUE,#

conf.int = TRUE,#

risk.table = F, #

#risk.table.col = "strata",

risk.table.y.text = F,

#linetype = "strata", #

#surv.median.line = "hv", #

xlab = "Time in years", #

xlim = c(0,max(res.cat$OS.time)+1),

break.time.by = 2, #

size = 1.5,

#ggtheme = theme_bw(),

palette = c("#2878b5", "#c82423")#

) + ggtitle("clinical_stage_I+II")

photo2 #

#

#

photo2$table <- photo2$table

# Changing the font size, style and color of photo2

# survival curves, risk table

photo2 #

}

##2.

your.km.plot("C16orf74")

III_IV <- clinical_stage[clinical_stage$clinical_stage == "III+IV",]

data <- III_IV %>%

dplyr::select(-clinical_stage) %>%

dplyr::select(fustat,futime,everything())

colnames(data)[1:2] <- c("OS","OS.time")

res.cut <- surv_cutpoint(data, time = "OS.time",

event = "OS",

variables = names(data)[3:ncol(data)],

minprop = 0.3) #

res.cat <- surv_categorize(res.cut)

summary(res.cut)

str(res.cat)

genes <- "C16orf74"

your.surv <- Surv(res.cat$OS.time, res.cat$OS)

your.km.plot <- function(genes,data){

print(genes)

group <- res.cat[,genes]

survival_dat <- data.frame(group = group)

group <- factor(group, levels = c("low", "high"))

fit <- survfit(your.surv ~ group)

sdf <- survdiff(your.surv ~ group,rho=0)

p.val <- 1 - pchisq(sdf$chisq, length(sdf$n)-1)

p.val

photo2 <- ggsurvplot(fit,data = survival_dat,

legend.title = genes,#

legend.labs = c("low","high"), #

#legend = "top",#

pval = T, #

#pval.method = TRUE,#

conf.int = TRUE,#

risk.table = F, #

#risk.table.col = "strata",

risk.table.y.text = F,

#linetype = "strata", #

#surv.median.line = "hv", #

xlab = "Time in years", #

xlim = c(0,max(res.cat$OS.time)+1),

break.time.by = 2, #

size = 1.5,

#ggtheme = theme_bw(),

palette = c("#2878b5", "#c82423")#

) + ggtitle("clinical_stage_III+IV")

photo2 #

#

#

photo2$table <- photo2$table

# Changing the font size, style and color of photo2

# survival curves, risk table

photo2 #

}

##2.

your.km.plot("C16orf74")
