# Supplementary material for: C16orf74 is a novel prognostic biomarker and associates with immune infiltration in head and neck squamous cell carcinoma
Source: PLoS One. 2025 May 7;20(5):e0322701. doi: 10.1371/journal.pone.0322701 (PMC12057912; doi:10.1371/journal.pone.0322701)
Supplement: S3 File — (ZIP) [file pone.0322701.s003.zip › Data Source.docx]

TCGA database: <https://portal.gdc.cancer.gov/>

GEO database: <https://www.ncbi.nlm.nih.gov/geo/>

TCGA-HNSC-count:

<https://gdc-hub.s3.us-east-1.amazonaws.com/download/TCGA-HNSC.star_counts.tsv.gz>

TCGA-HNSC-Survival:

<https://gdc-hub.s3.us-east-1.amazonaws.com/download/TCGA-HNSC.survival.tsv.gz>

TCGA-HNSC-Clinical:

<https://portal.gdc.cancer.gov>

GSE23358:

<https://www.ncbi.nlm.nih.gov/geo/query/acc.cgi?acc=GSE23558>

GSE30748:

<https://www.ncbi.nlm.nih.gov/geo/query/acc.cgi?acc=GSE30748>

GSE31056:

<https://www.ncbi.nlm.nih.gov/geo/query/acc.cgi?acc=GSE31056>

GSE184616:

<https://www.ncbi.nlm.nih.gov/geo/query/acc.cgi?acc=GSE184616>

GSE42743:

<https://www.ncbi.nlm.nih.gov/geo/query/acc.cgi?acc=GSE42743>

GSE145281 (IMvigor210 immunotherapy cohort):

<https://www.ncbi.nlm.nih.gov/geo/query/acc.cgi?acc=GSE145281>

<http://research-pub.gene.com/IMvigor210CoreBiologies/packageVersions/IMvigor210CoreBiologies_1.0.0.tar.gz>
